# FTO inhibition enhances the therapeutic index of radiation therapy in head and neck cancer

**DOI:** 10.1172/jci.insight.184968

**Published:** 2025-06-09

**Authors:** Lu Ji, Leighton Pu, Jinglong Wang, Hongbin Cao, Stavros Melemenidis, Subarna Sinha, Li Guan, Eyiwunmi E. Laseinde, Rie von Eyben, Sara A. Richter, Jin-Min Nam, Christina Kong, Kerriann M. Casey, Edward E. Graves, Richard L. Frock, Quynh Thu Le, Erinn B. Rankin

**Affiliations:** 1 Department of Radiation Oncology and; 2 Department of Computer Science, Stanford University, Stanford, California, USA.; 3 Division of Systemic Life Science, Graduate School of Biostudies, Kyoto University, Kyoto, Japan.; 4 Global Center for Biomedical Science and Engineering, Hokkaido University, Sapporo, Japan.; 5 Department of Pathology,; 6 Department of Comparative Medicine, and; 7 Department of Obstetrics and Gynecology, Stanford University, Stanford, California, USA.

**Keywords:** Oncology, Therapeutics, Head and neck cancer, Radiation therapy

## Abstract

Despite aggressive chemoradiation treatment, the overall survival rate for patients with HPV^–^ head and neck squamous cell carcinoma (HNSCC) remains poor, highlighting the urgent need for more effective drug-radiotherapy combinations to improve the therapeutic index of radiation therapy (RT). The fat mass and obesity-related gene (FTO) is emerging as a promising cancer therapeutic target; however, its role in the RT response has been underexplored. In our study, we found that both genetic and pharmacologic inhibition of FTO enhanced the efficacy of RT in human and mouse HNSCC tumor xenografts. Mechanistically, inhibition of FTO improved the RT response in HPV^–^ HNSCC cells, which was associated with increased DNA damage, reduced efficiency of homology directed repair, and decreased formation of RAD51 homolog 1 (RAD51) foci. Importantly, pharmacologic inhibition of FTO did not exacerbate radiation-induced oral mucositis, a significant normal-tissue toxicity associated with HNSCC RT. In summary, our results indicate a role for FTO in regulating homologous recombination while identifying FTO as a potential therapeutic target to enhance the therapeutic index of RT in HPV^–^ HNSCC treatment.

## Abstract 2

Targeting the m6A RNA demethylase FTO enhances the therapeutic index of radiation therapy in HPV-negative head and neck cancer.

## Introduction

Head and neck squamous cell carcinoma (HNSCC) is the sixth most common cancer worldwide (1). The majority (75%) of HNSCC are caused by tobacco use and have poorer outcomes compared with HNSCC associated with HPV infection (1). Approximately two-thirds of patients with HNSCC present with locoregionally advanced disease at the time of diagnosis and will require radiation therapy (RT) as a standard treatment (2). While RT is effective in a large proportion of patients, local failure remains a significant cause of patient morbidity and mortality (3). Systemic therapy, such as chemotherapy, can modestly improve local control and patient outcomes in HNSCC with a 6.5% increase in 5-year survival (2). However, for patients with locally advanced disease, toxicity limits the curative potential of chemoradiation (4).

The rational design of drug-radiotherapy combinations relies on exploiting the biological effects of radiotherapy within the tumor microenvironment. Tumor irradiation increases cancer cell oxidative stress, DNA damage, prosurvival signaling, and antigen release while reducing cancer cell proliferation and invasion (5). Therefore, therapeutic agents that modulate these pathways in the tumor microenvironment have the potential to enhance the radiation response of tumors (6). Current efforts are focused on developing targeted therapies that exploit these pathways in cancer to be utilized in combination with RT to improve patient outcomes. For example, targeted anti-EGFR therapy with cetuximab can improve overall survival in combination with radiotherapy in HPV^–^ in HNSCC with tolerable toxicity. However, the 5-year overall survival of cetuximab and radiotherapy is still only 46% compared with 36% with radiotherapy alone (7). Thus, additional targeted approaches that enhance the therapeutic index of radiotherapy are needed to improve HNSCC patient outcomes.

The RNA demethylase fat-mass and obesity-associated gene (FTO) is emerging as a new class of molecular targets for cancer therapy. FTO was discovered in 2011 as the first *N*^6^-methyladenosine (m^6^A) demethylase (8). m^6^A modifications are the most common internal mRNA modification present transcriptome-wide in at least 25% of all RNAs; they are reversible and dynamic, and they regulate gene expression and biological processes similar to DNA and histone modifications (9). FTO regulates pre-mRNA splicing, mRNA translation, degradation, and nuclear export (10, 11). Recent studies have shown that FTO is overexpressed and oncogenic in a variety of solid tumors (12–15). Functionally, FTO promotes cancer growth, survival, and modulation of the tumor immune microenvironment (15–21). In HNSCC, FTO is overexpressed, and high FTO expression correlates with reduced overall survival (22, 23). Although FTO is rapidly emerging as a cancer therapeutic target, few studies have characterized the role of FTO in the RT response or as a possible combination therapy to enhance the therapeutic index of RT (23, 24).

Here we sought to investigate the therapeutic potential of targeting FTO to enhance the therapeutic index of HPV^–^ HNSCC. Using both genetic and pharmacologic approaches, we demonstrate that inhibiting FTO enhances the radiation response of HNSCC tumors. Mechanistically, we demonstrate that FTO inhibition enhanced HNSCC cancer cell radiation response, which was associated with increased DNA damage, reduced homology directed DNA repair efficiency, and reduced RAD51 foci formation. Importantly, FTO inhibition did not exacerbate radiation-induced mucositis, an important normal-tissue toxicity associated with HNSCC radiotherapy. These findings underscore FTO as a molecular target for augmenting the therapeutic index of radiotherapy in HNSCC.

## Results

### Genetic and pharmacologic inhibition of FTO enhances HNSCC tumor radiation response.

**Figure 1:**
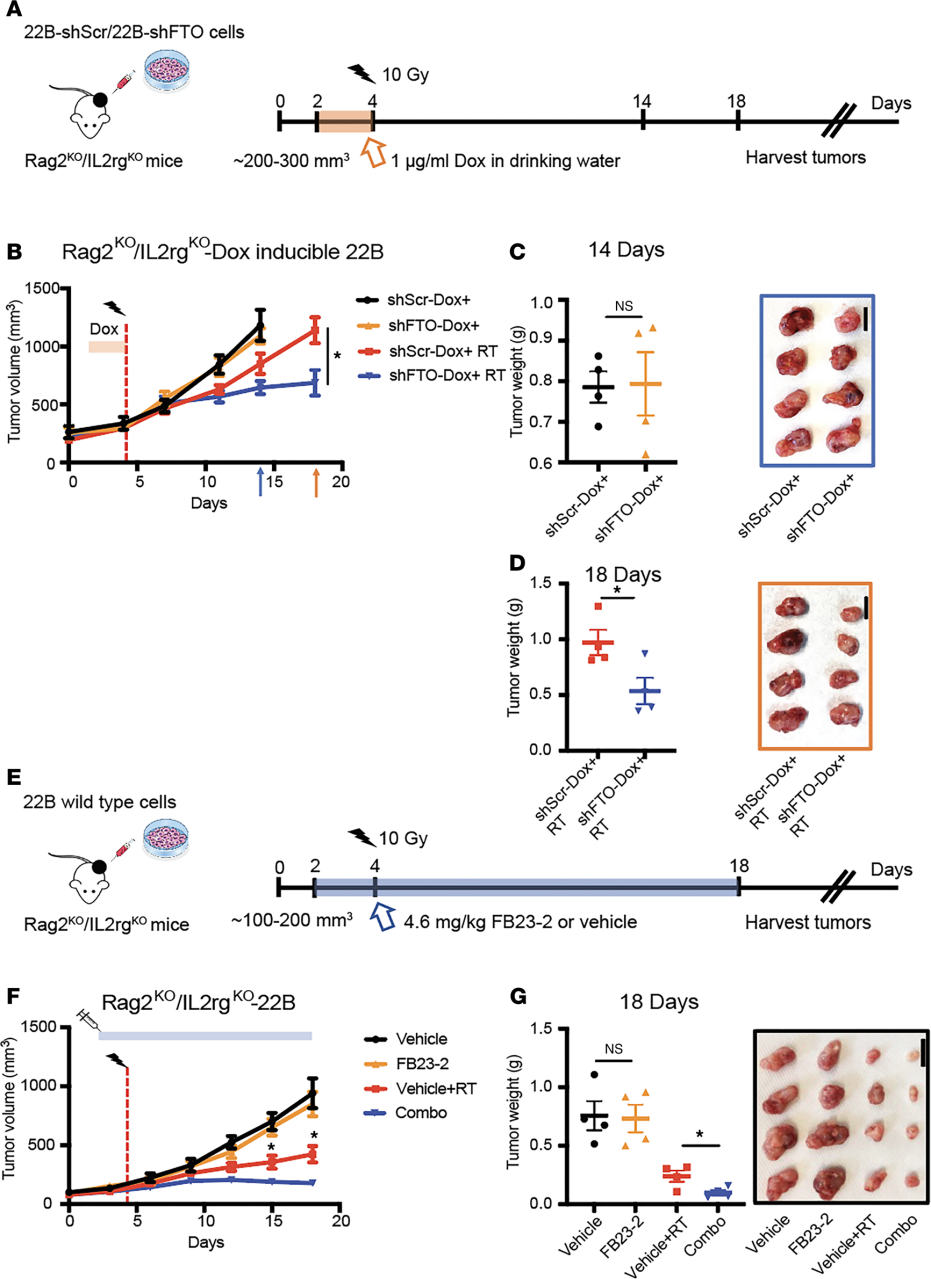
FTO inhibition enhances the radiation response of human HNSCC tumors. (**A**–**D**) Effect of genetic FTO inhibition on radiation response of UM-SCC-22B (22B) HNSCC cells in vivo. 22B doxycycline inducible control (shScr) or FTO knockdown (shFTO) cells were injected s.c. into male immunodeficient mice. When tumors reached ~200–300 mm^3^, mice treated with (1 μg/mL doxycycline in the drinking water) for 2 days before sham or 10 Gy irradiation. (**A** and **B**) Treatment schedule (**A**) and tumor growth curve (**B**) for the inducible control and FTO knockdown 22B tumor studies (*n* = 5–7 mice per group). Day 0 is the first day of tumor measurements. (**C** and **D**) Xenograft weights and representative xenograft images at the endpoint. (**E**–**G**) Effect of pharmacologic FTO inhibition on radiation response of 22B HNSCC cells in vivo. 22B cells were injected s.c. into male immunodeficient mice. When tumors reached approximately 100–200 mm^3^ mice treated with vehicle or the FTO inhibitor FB23-2 (4.6 mg/kg, i.p.) for 2 days before sham or 10 Gy irradiation. (**E**) Treatment schedule for the treatments in the 22B tumor studies. Day 0 is the first day of tumor measurements. (**F**) Tumor growth curves are shown (*n* = 7–8 mice per group). (**G**) Xenograft weights and representative xenograft images at the endpoint are shown. Arrows show the 14- and 18-day endpoints for the different comparison groups; the vertical dashed red line shows the time of irradiation; the orange box shows the scheme of doxycycline treatment; the blue box shows the timing of vehicle and FB23-2 treatment. Scale bar: 1 cm. *P < 0.05 as determined by 2-tailed *t* tests of the adjusted means from the generalized linear regression model. Data are presented as mean ± SEM.

Ideal candidates to radiosensitize tumors, but not normal tissues, include targets that are overexpressed in tumors relative to normal tissue. Previous studies have shown that FTO is overexpressed and high FTO expression correlates with reduced overall survival in patients with HNSCC (22, 23). In our independent analysis of the TCGA datasets, we confirmed that FTO expression is increased in HNSCC compared with normal adjacent tissue (Supplemental Figure 1; supplemental material available online with this article; https://doi.org/10.1172/jci.insight.184968DS1). To determine if FTO inhibition enhances HNSCC tumor radiation response, we generated shRNA-mediated FTO–knockdown (shFTO-knockdown) models in human HPV^–^ HNSCC cell lines (Supplemental Figure 2A). UM-SCC-22B cells harboring doxycycline-inducible shRNAs targeting a nontargeting control (shScr) or FTO (shFTO) were s.c. injected into the flanks of immunocompromised Rag2^ko^/IL2rg^ko^ mice. Once tumors reached approximately 200–300 mm^3^, the mice were randomized to receive either vehicle or doxycycline to induce shRNA expression (1 μg/mL Dox for 2 consecutive days in the drinking water) alone or in combination with RT (single fraction 10 Gy; Figure 1A). FTO knockdown in combination with RT reduced tumor growth compared with RT alone (Figure 1, B–D).

To determine the therapeutic potential of FTO demethylase inhibitors in this model, we next performed a tumor xenograft study using a potent and selective FTO demethylase inhibitor FB23-2 (13). When the tumors reached approximately 100–200 mm^3^, mice were treated with vehicle or FB23-2 (4.6 mg/kg) i.p. for 2 consecutive days before RT and daily following RT (Figure 1E). The ability of FB23-2 to inhibit FTO m^6^A RNA demethylase activity in this tumor model was confirmed by increased global m^6^A RNA levels in tumors isolated from mice treated with FB23-2 compared with tumors isolated from vehicle-treated mice (Supplemental Figure 2B). The combination treatment reduced tumor growth compared with RT alone (Figure 1, F and G).

**Figure 2:**
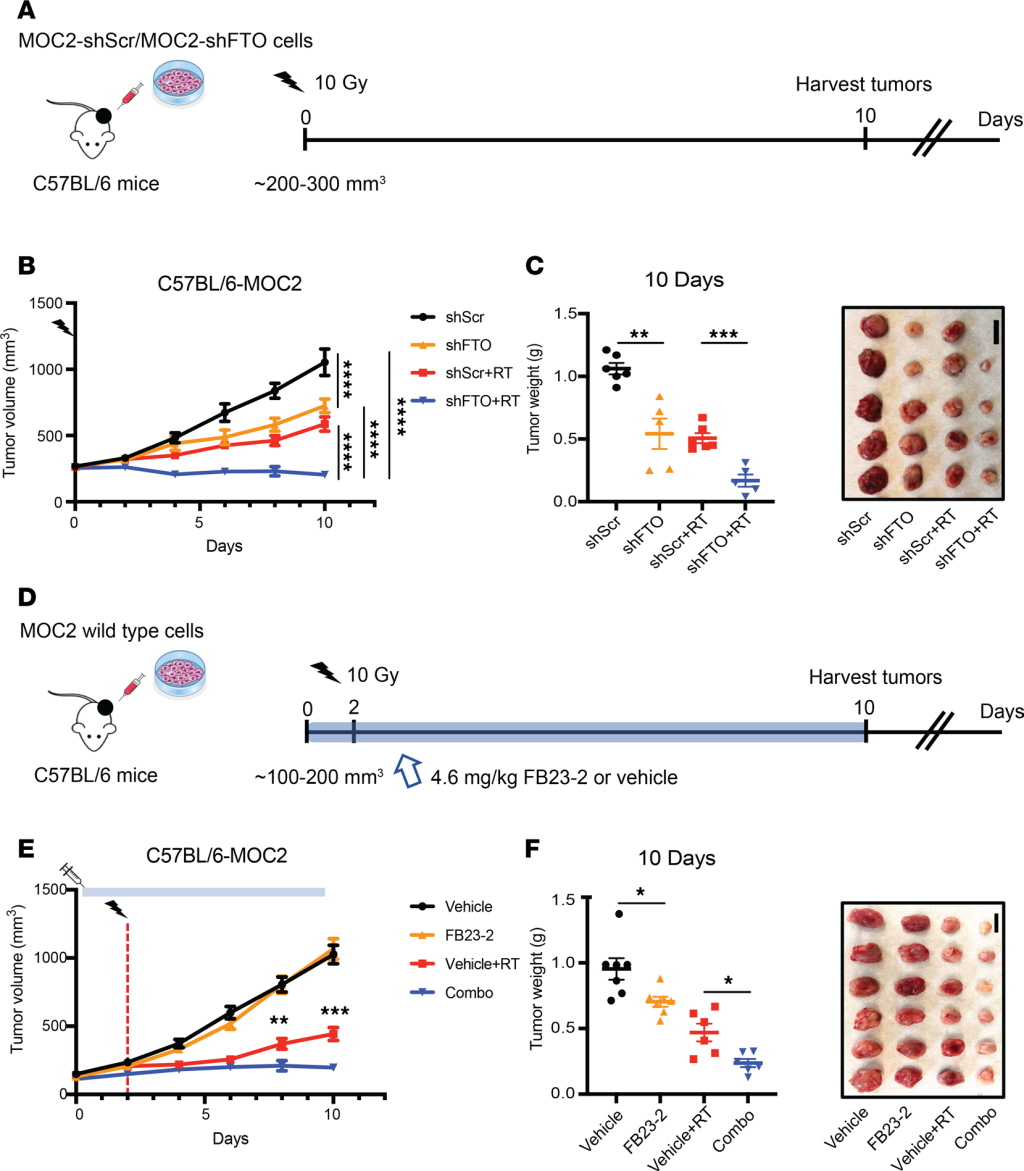
FTO inhibition enhances the radiation response of MOC2 murine HNSCC tumors. (**A**–**C**) Effect of genetic FTO inhibition on radiation response of murine MOC2 HNSCC cells in vivo. MOC2 stable control (shScr) or FTO knockdown (shFTO) cells were injected s.c. into male C57BL/6 mice. When tumors reached approximately 200–300 mm^3^, mice were treated with sham or 10 Gy irradiation. (**A**) Treatment schedule for the control and FTO knockdown MOC2 tumor studies. Day 0 is the time of irradiation. (**B**) Tumor growth curves are shown (*n* = 6–9 mice per group). (**C**) Xenograft weights (*n* = 5–6) and representative xenograft images at the endpoint. (**D**–**F**) Effect of pharmacologic FTO inhibition on radiation response of murine MOC2 HNSCC cells in vivo. MOC2 cells were injected s.c. into male C57BL/6 mice. When tumors reached approximately 100–200 mm^3^, mice were treated with vehicle or the FTO inhibitor FB23-2 (4.6 mg/kg, i.p.) for 2 days before sham or 10 Gy irradiation. (**D**) Treatment schedule for MOC2 treatment arms. (**E**) Tumor growth curves are shown (*n* = 12–13 mice per group). Day 0 is the first day of tumor measurements. The vertical dashed red line shows the time of irradiation; the blue box shows the timing of vehicle and FB23-2 treatment. (**F**) Xenograft weights (*n* = 6–7 per group) and representative xenograft images at the endpoint are shown. Scale bar: 1 cm. *P < 0.05, **P < 0.01, ***P < 0.001, ****P < 0.0001 as determined by 2-tailed *t* tests of the adjusted means from the generalized linear regression model. Data are presented as mean ± SEM.

Radiotherapy is effective not only by killing cancer cells but also through inducing antitumor immunity (25). Recent studies have revealed that genetic and pharmacologic inhibition of FTO not only affects cancer cell survival pathways but also can reprogram the immune response in the tumor microenvironment (17, 20). To determine the therapeutic potential of FTO inhibition in an immune-competent HNSCC murine model, we utilized the radiation-resistant MOC2 HPV^–^ HNSCC model (Supplemental Figure 3A). We first established stable control (shScr) and FTO knockdown (shFTO) MOC2 cell lines (Supplemental Figure 3B). Early-passage knockdown cells were then injected s.c. into the flanks of C57BL/6 mice. Once the tumors reached approximately 200–300 mm³, mice were treated with sham or 10 Gy single-fraction RT (Figure 2A). The combination of FTO knockdown with RT reduced tumor growth compared with control (shScr), FTO knockdown (shFTO), or RT alone (Figure 2, B and C). Interestingly, FTO knockdown alone also reduced tumor growth compared with control cells in the MOC2 model (Figure 2, B and C). Similar to genetic FTO knockdown, the combination treatment of FB23-2 and RT reduced tumor growth compared RT alone (Figure 2, D–F). Importantly, the vehicle- and FB23-2–treated mice had similar body weights in both sham and RT treatment groups (Supplemental Figure 4). Together, our findings demonstrate that genetic and pharmacologic inhibition of FTO enhances the radiation response in preclinical models of HPV^–^ HNSCC, indicating the therapeutic potential of FTO inhibitors as a strategy to radiosensitize HNSCC.

### FTO inhibition enhances the radiosensitivity of HPV^–^ HNSCC cells in vitro.

**Figure 3:**
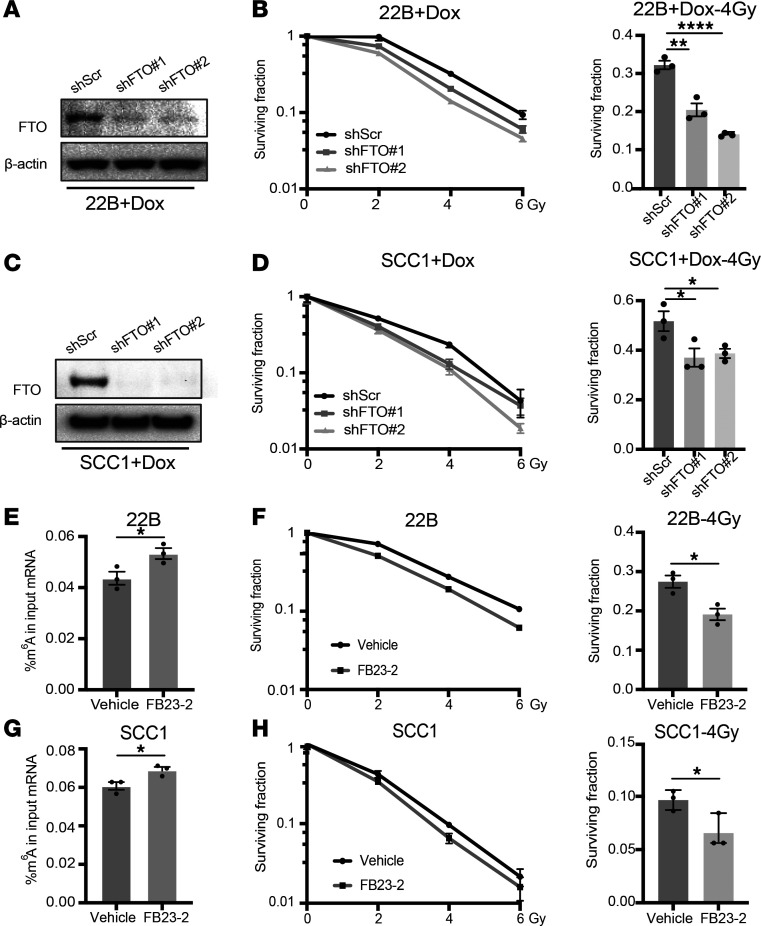
FTO inhibition enhances the radiosensitivity of HPV^–^ HNSCC cells. (**A**–**D**) Genetic inhibition of FTO enhances the radiation response on human HPV^–^ HNSCC cells. (**A** and **C**) Western blot analysis of FTO expression in doxycycline inducible control (shScr) and FTO knockdown (shFTO#1 and shFTO#2) UM-SCC-22B (**A**) and SCC1(**C**) cells 3 days after doxycycline treatment (2 μg/mL). β-Actin as used as a sample protein loading control. In **A**, the β-actin blot was set up in parallel and was run contemporaneously. (**B** and **D**) The effect of FTO knockdown on UM-SCC-22B (**B**) and SCC1 (**D**) clonogenic survival and radiation sensitivity (0, 2, 4, and 6 Gy; left panel). The surviving fraction is normalized to the corresponding control (0 Gy) and statistically compared with the control (shScr) group at 4 Gy (right panel, *n* = 3). (**E**–**H**) Pharmacologic inhibition of FTO enhances the radiation response on human HPV^–^ HNSCC cells. (**E** and **G**) Total m^6^A RNA analysis in FB23-2 treated UM-SCC-22B (**E**) and SCC1 (**G**) cells 24 hours after FB23-2 treatment (5 μM). (**F** and **H**) The effect of FB23-2 treatment on UM-SCC-22B (**F**) and SCC1 (**H**) clonogenic survival and radiation sensitivity (0, 2, 4, and 6 Gy; left panel). The surviving fraction is normalized to its control (0 Gy) and statistically compared with the control (vehicle) group at 4 Gy (right panel, *n* = 3). Data are presented as mean ± SEM; *P < 0.05, **P < 0.01, ****P < 0.0001 as determined by 2-tailed Student’s *t* test (**E**–**H**) and 2-way ANOVA (**B** and **D**).

To determine whether FTO inhibition alters the cancer cell intrinsic radiation response, we performed clonogenic survival assays in both the human (UM-SCC-22B and SCC1) and murine (MOC2) HPV^–^ HNSCC cell lines. First, we compared the clonogenic survival of UM-SCC-22B and SCC1 cells that contain a doxycycline-inducible shRNA nontargeting control or 2 independent doxycycline-inducible shFTO-knockdown lines (Figure 3, A–D). FTO knockdown in these cell lines enhanced the radiation response and decreased the fraction of surviving cells after 4 Gy radiation (Figure 3, B and D). We also generated stable control and FTO-knockdown cells with an shFTO-targeting sequence located within the 3′ UTR region of the gene enabling us to ectopically express either WT FTO or an FTO demethylase mutant (8). Ectopic expression of WT FTO, but not the demethylase mutant FTO, enhanced the radiation response in the FTO knockdown UM-SCC-22B cells (Supplemental Figure 5, A and B). Finally, we used a SMARTpool siRNA approach, which combines 4 gene-specific siRNAs into a single-reagent pool, to knockdown FTO expression in UM-SCC-22B and SCC1 cells. Similarly, FTO knockdown using siRNA approaches enhanced the radiation response of HNSCC cell lines (Supplemental Figure 6, A–D). Overall, these data demonstrate that genetic inhibition of FTO enhances the radiation response of human HNSCC cells.

To determine whether pharmacologic inhibition of FTO demethylase activity with FB23-2 is sufficient to enhance the radiation response of HPV^–^ HNSCC cells, we treated the UM-SCC-22B and SCC1 cells with FB23-2 (5 μM) for 24 hours before irradiation. FB23-2 treatment increased the global m^6^A RNA levels and enhanced the radiation response in both cell lines (Figure 3, E–H). Similar results were observed in the murine MOC2 cell line where genetic and pharmacologic inhibition of FTO enhanced the cancer cell radiation response (Supplemental Figure 7, A–D). FTO inhibition at baseline (0 Gy) did not influence the survival of the HNSCC cell lines utilized in this study (Supplemental Figure 8, A and B). Together, FTO inhibition enhanced the radiation response of HNSCC cells in vitro, suggesting that the efficacy of FTO inhibition in vivo is mediated, at least in part, through cancer cell intrinsic mechanisms affecting cancer cell radiosensitivity.

### FTO inhibition in combination with radiation results in persistent DNA damage in HNSCC cells.

**Figure 4:**
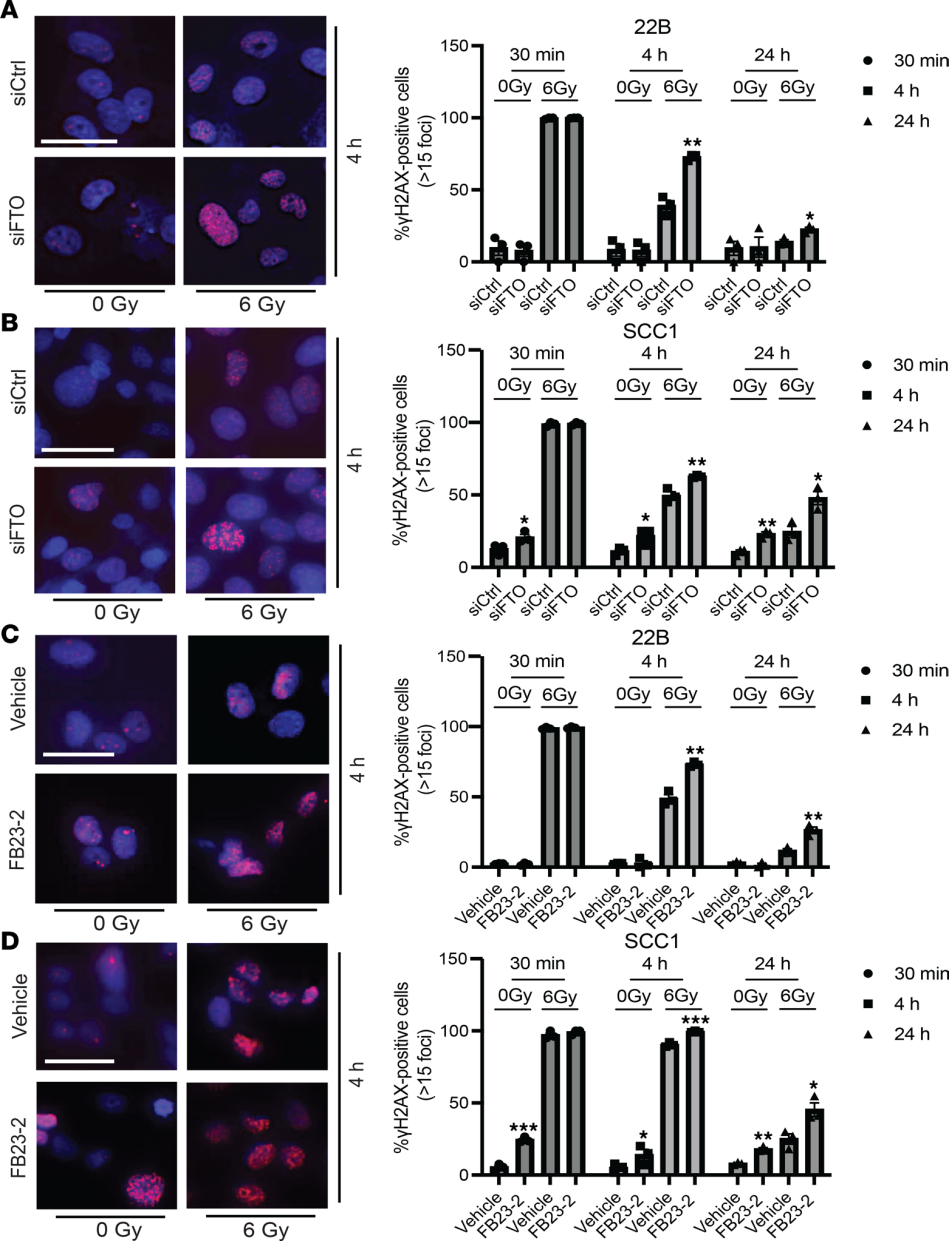
FTO inhibition results in persistent DNA damage in HPV^–^ HNSCC cells after irradiation. (**A** and **B**) Genetic inhibition of FTO increases γH2AX foci at 4 and 24 hours after irradiation in human HPV^–^ HNSCC cells. **A** and **B** show representative images (at 4 hours after 0 or 6 Gy irradiation) and quantification of γH2AX foci (red) immunofluorescence staining (30 minutes, 4 hours, and 24 hours after irradiation) in siRNA (SMARTpool) control and FTO knockdown UM-SCC-22B (**A**) and SCC1 (**B**) cells. (**C** and **D**) Pharmacologic inhibition of FTO increases γH2AX foci at 4 and 24 hours after irradiation in human HPV^–^ HNSCC cells. **C** and **D** show representative images at 4 hours after 0 or 6 Gy irradiation and quantification of γH2AX foci (red) immunofluorescence staining (30 minutes, 4 hours, and 24 hours after irradiation) in vehicle and FB23-2 (5 μM) treated UM-SCC-22B (**C**) and SCC1 (**D**) cells. Quantification of the percentage of cells with > 15 γH2AX foci per cell is based on 3 random fields and 30 cells from each biologic replicate in each group (*n* = 3). Each dot represents the average for an individual biologic replicate in each group. Nuclei were stained with DAPI (blue). Scale bars: 25 μm. *P < 0.05; **P < 0.01; ***P < 0.001 as determined by 2-tailed Student’s *t* test and 2-way ANOVA. Data are presented as mean ± SEM.

To investigate the cancer cell intrinsic mechanisms by which FTO inhibition enhances HNSCC radiation response, we next investigated whether FTO inhibition affects DNA damage levels in irradiated HNSCC cells. We examined the effect of FTO inhibition on DNA breaks by quantifying γH2AX foci, a marker of unresolved DNA damage, in HPV^–^ HNSCC cells treated with control siRNA, FTO siRNA, RT alone, or the combination of FTO siRNA and RT over time. At 30 minutes after RT, we detected extensive γH2AX foci formation in both control and FTO knockdown cells (Figure 4, A and B). At the 4- and 24-hour time points after RT, the percentage of cells with > 15 γH2AX foci declined in both siControl and siFTO knockdown cells. However, we observed an increase in the percentage of cells with > 15 γH2AX foci in the FTO knockdown cells compared with the control cells (Figure 4, A and B; Supplemental Figure 9, A and B; and Supplemental Figure 10A). Similarly, FTO inhibition with FB23-2 increased the percentage of cells with > 15 γH2AX foci at the 4- and 24-hour time points after RT in all cell lines (Figure 4, C and D; Supplemental Figure 9, A and B; and Supplemental Figure 10B). We also observed that FTO inhibition in SCC1 and MOC2 cells increased the percentage of cells with > 15 γH2AX foci at 0 Gy (Figure 4, B and D, and Supplemental Figure 10, A and B). Overall, these data demonstrate that FTO inhibition increases DNA damage in HNSCC cells.

### FTO inhibition decreases homology-directed repair and RAD51 foci formation.

**Figure 5:**
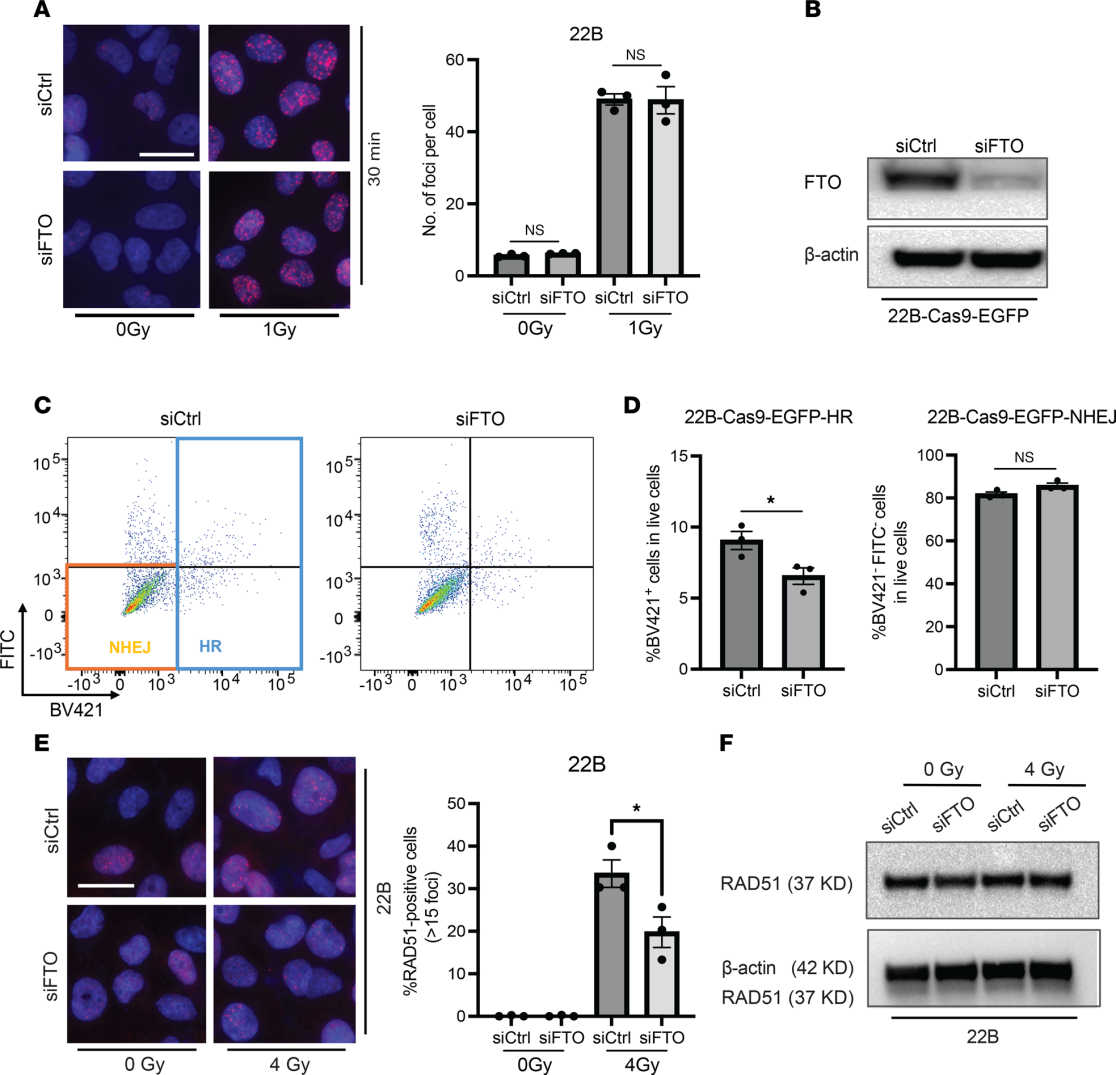
FTO knockdown reduces the efficiency of homologous recombination in human HPV^–^ HNSCC cells. (**A**) FTO knockdown does not increase γH2AX foci at 30 minutes after irradiation in human HPV^–^ HNSCC cells. A shows representative images at 30 minutes after 0 or 1 Gy irradiation and quantification of γH2AX foci (red) immunofluorescence staining in siRNA (SMARTpool) control and FTO knockdown UM-SCC-22B cells. Quantification of the total number of γH2AX foci per cell based on 3 random fields and 15 cells from each biologic replicate in each group (*n* = 3). Each dot represents the average for an individual biologic replicate in each group. Nuclei were stained with DAPI (blue). Scale bar: 25 μm. (**B**–**D**) FTO knockdown reduces the efficiency of homology directed repair in human HPV^–^ HNSCC cells. (**B**) Western blot analysis of FTO expression in siRNA control (siCtrl) and FTO knockdown (siFTO) UM-SCC-22B cells with stable EGFP expression. (**C**) Representative FACS plot analysis of EGFP (FITC) and BFP donor (BV421) expression in siRNA control (siCtrl) and FTO knockdown (siFTO) UM-SCC-22B cells nucleofected with EGFP-sgRNA and Cas9 expression. (**D**) The percentage of live BV421^+^ homology–directed repair positive (HR) or FITC^–^BV421^–^ nonhomologous end joining (NHEJ) control (siCtrl) or FTO knockdown (siFTO) UM-SCC-22B cells. Each group is statistically compared with siRNA control group (*n* = 3). (**E**) FTO knockdown reduces RAD51 foci formation in irradiated human HPV^–^ HNSCC cells. Left panel shows representative images of RAD51 foci (red) formation in siRNA control (siCtrl) and FTO knockdown (siFTO) UM-SCC-22B cells at 4 hours after irradiation. Right panel shows quantification of the percentage of cells with > 15 RAD51 foci per cell based on 3 random fields and 30 cells from each biologic replicate in each group (*n* = 3). Each dot represents the average for an individual biologic replicate in each group. Nuclei were stained with DAPI (blue). (**F**) Western blot analysis of RAD51 protein levels in siRNA control (siCtrl) and FTO knockdown (siFTO) in UM-SCC-22B cells 4 hours after 0 Gy or 4 Gy irradiation. Scale bar: 25 μm. *P < 0.05 as determined by 2-tailed Student’s *t* test (**A** and **D**) and 2-way ANOVA (**E**). Data are presented as mean ± SEM.

Our data above demonstrate that FTO inhibition combined with radiation results in increased persistent DNA damage, potentially due to either increased initial DNA damage and/or impaired DNA repair. To investigate whether FTO inhibition results in increased initial DNA damage at early time points after irradiation, we reduced the radiation dose to 1 Gy and compared the total number of γH2AX foci in control and FTO knockdown cells at 30 minutes after irradiation. The number of γH2AX foci in control and FTO knockdown cells were similar at 30 minutes after irradiation, suggesting that the persistent DNA damage observed in FTO-knockdown cells may be not due to increased initial DNA damage after RT (Figure 5A).

Based on these findings, we hypothesized that the persistent DNA damage in FTO knockdown cells after irradiation may be due to reduced DNA repair efficiency. Homologous recombination (HR) and nonhomologous end joining (NHEJ) are the 2 primary pathways of DNA double-strand break (DSB) repair (26). To determine whether FTO affects these pathways in HNSCC cells, we utilized an established DNA DSB repair assay that utilizes the traffic light reporter (TLR) for the simultaneous quantification of homology directed repair and NHEJ activity following a targeted CRISPR/Cas9-mediated DNA break in the EGFP gene (27). FTO knockdown reduced efficiency of homology directed repair but did not affect the efficiency of NHEJ in the UM-SCC-22B cells (Figure 5, B–D). To further explore mechanisms underlying the reduced efficiency of homology directed repair in FTO knockdown cells, we compared RAD51 foci formation, a marker of active HR repair (28), in the control and FTO knockdown HNSCC cancer cells before and after RT. FTO inhibition reduced RAD51 foci formation at 4 hours after irradiation in the UM-SCC-22B cells (Figure 5E). A similar reduction in RAD51 foci formation was observed in FB23-2 treated UM-SCC-22B cells at 4 hours post irradiation (Supplemental Figure 11A). Moreover, we observed a similar reduction of RAD51 foci formation in SCC1 FTO knockdown and FB23-2 treated cells (Supplemental Figure 11, B and C). Western blot analyses revealed that RAD51 protein levels were comparable across all treatments, suggesting that the decrease in RAD51 foci formation could not be attributed to a general reduction in RAD51 protein levels following FTO inhibition (Figure 5F and Supplemental Figure 11, D–F). Overall, these data demonstrate that FTO inhibition impairs homology directed repair efficiency and RAD51 foci formation and promotes DNA damage in HNSCC cells.

### FTO inhibition does not affect the severity of radiation-induced oral mucositis.

**Figure 6:**
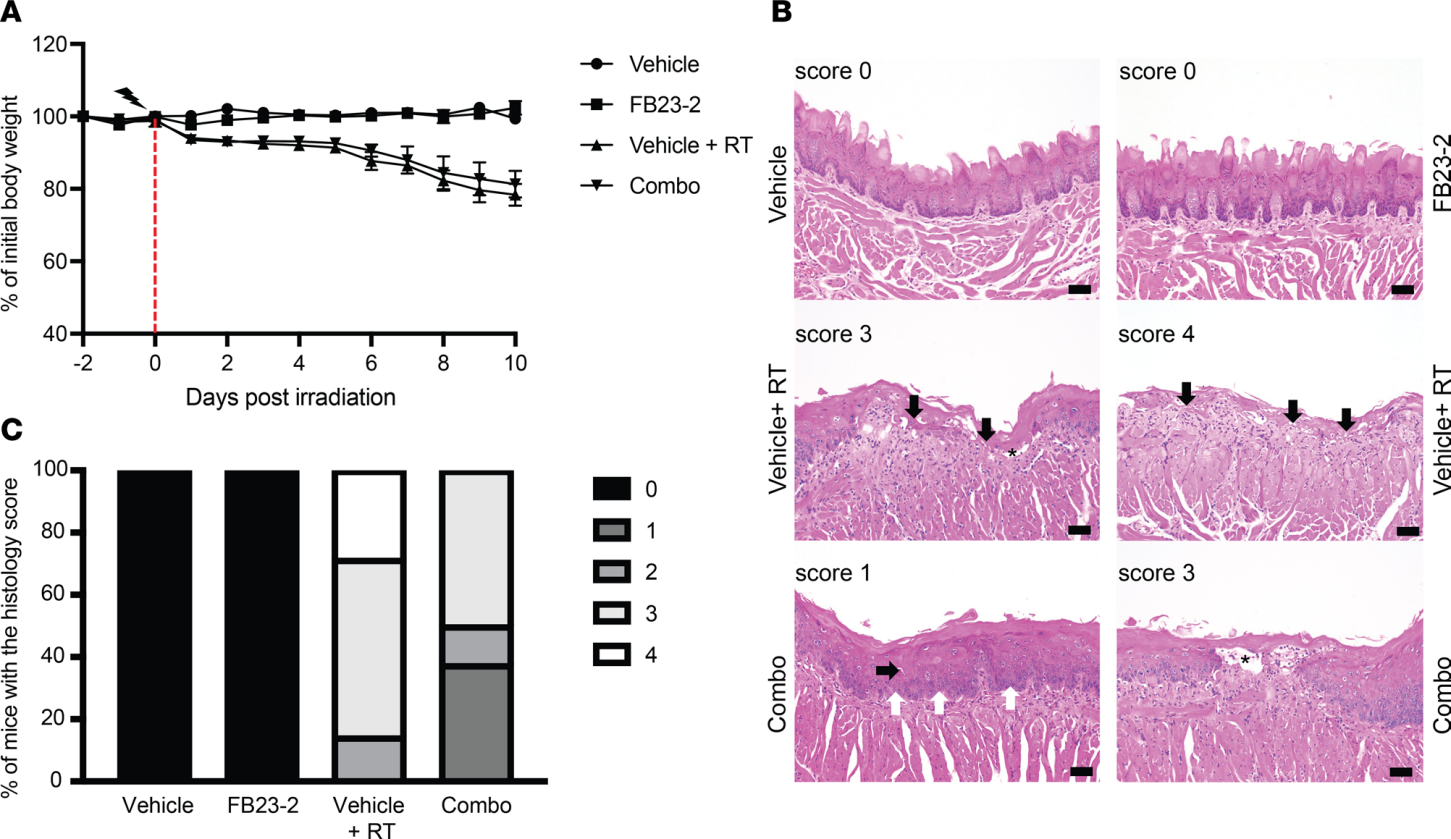
FTO inhibition does not increase radiation-induced oral mucositis. (**A**) Percentage of individual mouse body weights following treatment with vehicle or FB23-2 (4.6 mg/kg i.p. daily) alone and in combination with single fraction 18 Gy irradiation. Vehicle or FB23-2 treatments began 2 days before irradiation and continued for 10 days after irradiation. Vertical dashed red line shows the day of irradiation. (**B**) Representative images of H&E-stained tongue sections at 10 days after irradiation. Score 0 in unirradiated vehicle and FB23-2 treated mice show normal organization of the stratified squamous epithelium. Mouse tongues receiving a score of 1 exhibit disorganization of the stratum basal (white arrows) and occasional dyskeratotic epithelial cells (black arrows); score 2 shows epithelial thinning or the presence of 3 or more dyskeratotic squamous cells within the epithelium; score of 3 exhibit bulla formation (asterisk) and loss of epithelium without a break in keratinization (black arrows); and score of 4 exhibit ulceration of the epithelium (black arrows). Scale bar: 50 μm. (**C**) Graph showing the percentage of mice in the vehicle (*n* = 7), FB23-2 (*n* = 7), vehicle + RT (*n* = 7), and FB23-2 + RT (*n* = 8) that had the highest score of 0, 1, 2, 3, or 4.

Our results above suggest that FTO inhibition may be an effective strategy to enhance the radiation response of HNSCC tumors. To determine the safety of this combination therapy, we investigated the normal tissue effects of FTO inhibitors in combination with radiation within the oral mucosa. In these studies, we focused on radiation-induced mucositis to the tongue as radiation-induced oral mucositis (RIOM) is a dose-limiting toxicity in patients with HNSCC that is characterized by oral pain, dysphagia, opioid use, and weight loss (29). The head and neck region of C57BL/6 mice was irradiated with a single fraction of 18 Gy. Both vehicle and FB23-2 irradiated groups lost body weight at a similar rate for 10 days post irradiation (Figure 6A). At day 10 post RT, the mice were euthanized and the tongues of the control and FB23-2 irradiated groups were scored for oral mucositis using a standardized scoring system (30). The percentage of mice with a grade 3 or 4 oral mucositis score was 85.7% in the mice treated with vehicle and RT compared with 50% in the mice treated with FB23-2 and RT (Figure 6, B and C). Importantly, these data indicate that FB23-2 treatment does not enhance RIOM and may reduce the severity of RIOM.

## Discussion

Our findings identify FTO as an important epitranscriptomic factor and therapeutic target to augment the therapeutic index of radiotherapy in HNSCC. FTO is well characterized in its role in promoting cancer growth and survival. It has been shown to promote cancer cell growth and survival pathways through the regulation of MYC and BNIP3 (17–19). FTO can also reprogram the tumor immune microenvironment by suppressing immune checkpoint gene expression (PD-L1, PD-L2) and modulating metabolic cancer cell-T cell nutrient competition (17, 20, 21). Multiple studies have reported that FTO inhibitors improve responses to immune checkpoint therapy (17, 20, 21). Although FTO is rapidly emerging as a therapeutic target for cancer therapy, few studies have evaluated the therapeutic potential of targeting FTO to enhance the therapeutic index of radiotherapy. A recent study demonstrated that FTO inhibition with FB23-2 enhances the efficacy of radiotherapy in EBV-positive radioresistant nasopharyngeal carcinoma cells and tumors (23). Zhou et al. reported that FTO overexpression promotes chemo-radiotherapy resistance in cervical cancer cells and tumors (24). The role of FTO inhibition on the normal tissue response to radiotherapy has not been investigated. We show that both genetic and pharmacologic inhibition of FTO enhances the sensitivity of HPV^–^ HNSCC to RT, both in vitro and in vivo. Moreover, pharmacologic inhibition of FTO does not exacerbate radiation-induced mucositis, a significant normal tissue toxicity associated with HNSCC radiotherapy.

There is growing evidence to support a functional role for FTO in DNA damage repair. m^6^A RNA levels increase at sites of DNA damage following ultraviolet (UV) irradiation, and notably, FTO localizes to these damage sites where it reduces m^6^A RNA levels and the persistence of these modifications after UV exposure (31). Functionally, FTO knockout osteoblasts are more susceptible to UV-induced damage and apoptosis (32). UV-induced DNA damage, along with lesions caused by chemical agents and oxidative stress, are repaired through the nucleotide excision repair (NER) pathway (33). Emerging evidence suggests that FTO plays a role in promoting nucleotide excision repair. For example, FTO overexpression in cervical cancer cells enhances chemo-radiation resistance, which correlates with decreased expression of the excision repair cross-complementation group 1 (ERCC1) protein, a crucial factor in nucleotide excision repair (24). Additionally, FTO promotes the expression of ERCC1 and other NER genes, including POLD3 and RFC2, in human lymphoblastoid cells (34).

Regarding DNA DSB repair, FTO promotes proliferation and regulates DNA damage in renal cell carcinoma cells through the expression of DNA polymerase θ (POLQ), a key protein involved in microhomology-mediated end joining (35). Our data implicate a role for FTO in HR. Both genetic and pharmacologic inhibition of FTO increased the radiosensitivity of HNSCC cells, which was associated with increased DNA damage, reduced efficiency of homology-directed DNA repair, and decreased RAD51 foci formation. These findings pave the way for future research investigating how FTO regulates RAD51 foci formation and homologous recombination in HNSCC cells.

Finally, our findings indicate that therapeutic targeting of FTO may be an effective strategy to enhance the therapeutic index of RT. We demonstrate that pharmacologic inhibition of FTO demethylase activity with a small molecule is sufficient to enhance the efficacy of RT in the treatment of human and mouse HNSCC tumor xenografts. Importantly, pharmacologic inhibition of FTO did not increase radiation-induced mucositis in the tongue, suggesting that small molecule FTO inhibitors have the potential to enhance the therapeutic index of radiotherapy in HNSCC. While current inhibitors serve as important tool compounds to study FTO activity, the clinical activity of these drugs are still limited due to poor pharmacodynamics. Since clinical FTO inhibitors are being developed for the treatment of obesity and cancer, this study provides the preclinical data to support future studies evaluating the efficacy and safety of clinical FTO inhibitors as radiosensitizers.

## Methods

### Sex as a biological variable.

Our study was performed in male mice due to the increased incidence of HNSCC in males compared with females. Men have a 2- to 4-fold higher risk of developing HNSCC compared with women (1).

### FTO TCGA analysis.

Tumor versus normal differential FTO expression analysis for TCGA HNSCC tumor, versus normal adjacent, differential analysis of FTO gene expression in tumor, versus normal samples was performed using Wilcoxon rank sum test.

### Cell culture.

Human HPV^–^ HNSCC UM-SCC-22B (36) and SCC1 (37) cells were obtained from MilliporeSigma and cultured in DMEM containing 10% FCS. The HPV^–^ murine oral cancer cells (MOC1 and MOC2) (38) were obtained from Ravindra Uppaluri (Dana Farber Cancer Institute, Boston, Massachusetts, USA) and were cultured in IMDM and Ham’s F-12 medium (2:1) with 5% FCS, 40 ng/mL hydrocortisone (Sigma-Aldrich), 5 ng/mL EGF (Sigma-Aldrich), 5 μg/mL insulin (Sigma-Aldrich), and 1% penicillin-streptomycin (Sigma-Aldrich). The HPV^+^ murine oral cancer cells (mEERL; mouse E6/E7 and H-Ras transformed luciferase expressing mouse tonsil epithelial cells) (39) were obtained from William Spanos (Sanford Research, Sioux Falls, South Dakota, USA) and were grown in DMEM/Ham’s F-12 (2:1), containing 10% FBS, 25 μg/mL hydrocortisone, 5 μg/mL transferrin, 5 μg/mL insulin, 1.36 ng/mL triiodothyronine, and 5 ng/mL EGF. All cells were tested for viability, cell morphology, and the presence of mycoplasma and viruses. All cells were cultured at 37°C supplied with 5% CO_2_.

### siRNA and plasmid transfections.

SMARTpool siRNAs targeting control, human FTO, and mouse FTO were ordered from Horizon Discovery (Supplemental Table 1). siRNAs were transfected into cells using Lipofectamine RNAiMax (Invitrogen) with Opti-MEM media (Invitrogen) according to the manufacturer’s instructions.

Mouse shFTO plasmids were ordered from MilliporeSigma and are listed in Supplemental Table 2. Dox-inducible human shFTO plasmids and Dox-inducible nontargeting shScr plasmid were purchased from Dharmacon (V3SH11252-227508210, VSC11653). For the rescue experiments, we utilized stable shScr and shFTO constructs that were previously described (16). The shRNA targeting sequences are listed in Supplemental Table 2. For the generation of lentiviral particles, 293T cells were transfected by a cocktail of 1.5 μg shFTO or nontargeting control constructs, packaging vectors (0.5 μg of VSVG [pCMV-VSV-G] and 2 μg of Δ8.2 [pCMV-dR8.2 dvpr]) and Lipofectamine 3000 (Thermo Fisher Scientific). The lentiviral supernatants were harvested at 48 and 72 hours after transfection using 0.45 μm filter and added to target HNSCC cells with 5 μg/mL polybrene. The lentiviral infected HNSCC cells were then selected with puromycin (2.5 mg/mL) for 5 days or until control cells without infection were all dead.

### Clonogenic survival assay.

To inhibit FTO in HNSCC cells, UM-SCC-22B, SCC1, and MOC2 cells were pretreated with 5 μM FB23-2 (Sigma-Aldrich, SML2694-25G) for 24 hours before irradiation (0, 2, 4, 6 Gy). The drug was not present in the cell culture media following the 24-hour pretreatment. To induce FTO knockdown, the cells were treated with 2 μg/mL Dox (Sigma-Aldrich, D9891-5G) for 2 days, siRNA for 2–3 days, or stable plasmids transfection before irradiation (0, 2, 4, 6 Gy). The cells were plated in triplicate in 6-well plates at different densities ranging from 400 to 5,000 cells per well. After 10–14 days, the cells were stained with 0.05% crystal violet. The surviving fraction (SF) was determined by counting the number of colonies with more than 50 cells. The cell plating efficiency (PE) was determined by survival of unirradiated cells. A SF was normalized by the PE: PE = (no. of colonies formed) / (no. of cell seeded) × 100%; SF = (no. of colonies formed after treatment) / (no. of cells seeded) / PE.

### Immunofluorescence staining for γH2AX and RAD51 foci.

UM-SCC-22B, SCC1, and MOC2 cells were pretreated with 5 μM FB23-2 for 24 hours, and siRNAs were pretreated for 2–3 days before irradiation (4 Gy or 6 Gy). The cells were fixed by 4% paraformaldehyde in PBS (pH 7.4) for 10 minutes at room temperature; then, the cells were permeabilized with 0.3% Triton X-100 for 10 minutes and incubated cells with 2% BSA for 30 minutes. The cells were incubated with anti-γH2AX antibody or anti-RAD51 antibody overnight at 4°C, followed by staining with the goat anti-rabbit IgG (H+L) Alexa Fluor 594–labeled secondary antibody at room temperature for 1 hour. DAPI (Sigma-Aldrich) was used for nuclear counterstaining at 0.1 μg/mL, and representative images were taken under the Leica SPE microscope. Cells with more than 15 γH2AX foci or RAD51 foci were scored as positive. The list of antibodies and dilutions used is provided in Supplemental Table 3.

### DNA DSB assay.

The DSB assay was conducted as previously described, with the following modifications (27, 40). Briefly, UM-SCC-22B cell lines were transduced with lentivirus carrying the pCDH plasmid encoding the EGFP-Puro gene. Cells were selected in DMEM containing 5 μg/mL puromycin for approximately 1 week until > 99% of the cells were EGFP^+^. Two days prior to nucleofection, UM-SCC-22B cells were seeded in 6-well plates at 25% confluence. siRNAs targeting FTO and a nontargeting control (siCtrl) were transfected using Lipofectamine RNAiMax (Invitrogen) with Opti-MEM media (Invitrogen) according to the manufacturer’s instruction. After 24 hours, the siRNA-containing medium was replaced with fresh DMEM supplemented with 200 ng/mL Nocodazole (Selleckchem, S2775) for cell cycle synchronization. Following 20 hours of Nocodazole treatment, both adherent cells and detached cells (floating in the medium) were harvested and pooled for nucleofection. For each nucleofection, 50 pmol of EGFP-sgRNA (spacer sequence: ctcgtgaccaccctgaccta) was preincubated with 50 pmol Cas9 protein (IDT, 1081058) at room temperature for 20 minutes to form Cas9-RNP complexes. The Cas9-RNP complexes and 50 pmol single-strand BFP donor template were mixed with 100 μL of transfection buffer (Lonza, V4XC-2024) and used to resuspend the cell pellet. The nucleofection mixture was transferred to a nucleofection cuvette and subjected to nucleofection using the FF-120 program on a Lonza 4D-Nucleofector. After nucleofection, cells were cultured in fresh DMEM for approximately 1 week. Flow cytometry was performed to analyze the expression of EGFP and BFP. Oligonucleotides used in this study are provided in Supplemental Table 4.

### Western blot.

For protein expression analysis, whole cell protein was isolated in urea lysis buffer and processed for protein loading as previously described (16). The antibodies and dilution used for Western blot are listed in Supplemental Table 3.

### mRNA extraction and quantitative PCR (qPCR).

Total RNA was isolated with TRIzol reagent (15596026, Invitrogen) according to the manufacturer’s instructions. cDNA synthesis was performed using Iscript cDNA Synthesis Kit (1708891, Bio-Rad). Real-time PCRs were performed using Itaq Universal SYBR (1725124, Bio-Rad). Reactions were run and analyzed using ABI-7900HT Fast Real-Time PCR System (Applied Biosystems), and relative quantification was normalized to the amount of 18S. Primers used for real-time PCR are listed in Supplemental Table 4.

### Mouse xenograft studies.

To establish HNSCC xenografts, Rag2^ko^IL2rg^ko^ male mice aged from 6 to 8 weeks were injected with 1 × 10^7^ UM-SCC-22B cells s.c. in the flank region. Similarly, 6- to 8-week-old, male C57BL/6 mice (strain no. 000664; RRID: IMSR_ JAX: 000664) were purchased from The Jackson Laboratory. For MOC2 tumor experiments, mice were injected s.c. in the flank region (2.4 × 10^5^). Primary tumors were measured using a digital caliper 2–4 times per week, and mice were monitored for tumor growth over time. Mice were sacrificed when the tumor reached approximately 1,000 mm^3^. Tumor volume = (length × width^2^)/2.

### Mouse treatments.

When the tumor volume reached approximately 100–200 mm^3^, 4.6 mg/kg FB23-2 or vehicle were administrated i.p. daily until the end of the mouse study. Radiation was administered in a dose of 10 Gy at 2 days after initial FB23-2 treatment. For stable FTO knockdown MOC2 tumors, 10 Gy radiation was administrated when the tumor volume reached approximately 200–300 mm^3^. For Dox-inducible FTO–knockdown UM-SCC-22B tumors, 1 μg/mL Dox was administrated through drinking water to induce FTO knockdown when tumors reached approximately 200–300 mm^3^ at 2 days prior to RT. For m^6^A ELISA in vivo, we conducted a mouse study using UM-SCC-22B tumor–bearing mice treated with either vehicle or 4.6 mg/kg FB23-2 for 5 consecutive days. On day 5, tumors were collected 4 hours after the final FB23-2 treatment. mRNA was extracted and analyzed using m^6^A ELISA.

### Histopathological scoring of radiation-induced mucositis.

Histopathologic scoring of oral mucositis was performed according to the criteria outlined by Sunavala-Dossabhoy et al. (30). The grading system ranged from 0 to 4, with each grade reflecting varying degrees of disease severity. Briefly, grade 0 indicated the absence of radiation injury, with the mucosa appearing normal. Grade 1 represented either focal or diffuse alterations in the basal cell layer, characterized by nuclear atypia and the presence of up to 2 dyskeratotic squamous cells, and/or disorganization of squamous differentiation. Grade 2 denoted epithelial thinning or the presence of 3 or more dyskeratotic squamous cells within the epithelium. Grade 3 indicated the loss of epithelium without a break in keratinization, the presence of atrophied eosinophilic epithelium, or subepithelial vesicles. Grade 4 was indicative of loss of epithelial and keratinized cell layers, accompanied by ulceration.

A total of 6 cross-sectional images from each tongue were evaluated and scored independently and blinded by a veterinary pathologist. Lesion grades were assigned to each cross-sectional image based on the region with the highest severity. Thus, each cross-sectional image received one grade.

### Irradiation.

Cells were irradiated using a 225 kVp cabinet x-ray irradiator filtered with 0.5 mm Cu (IC-250, Kimtron Inc.).

For the s.c. tumor and head and neck irradiation on mice, RT was delivered using the PXi X-Rad SmART (Precision X-ray Inc.) with a radiation source of a 225 kV x-ray tube filtered by 0.3 mm Cu, operated at 13 mA. Mice were irradiated at a dose rate of 3.35 Gy/min (335 rad/min) at isocenter from an uncollimated beam, a distance of 30 cm source to subject distance (SSD).

Dosimetry calibration was performed using a PTW Farmer Ionization chamber to measure exposure in air, and the dose rate was calculated using AAPM report TG-61 (41). This was done at the standard SSD of 30. Radiochromic film dosimetry was used to measure collimator output factors and penumbras. Depth dose curves in water were measured by placing films in between sheets of solid water to measure doses at depths up to 3 cm.

Mice were placed on the sample bed and anesthetized with isoflurane through a nose cone. Cone beam CT images were acquired and reconstructed, from which a treatment plan was created. The treatment plan consisted of a series of collimated 225 kV, 13 mA beams directed at the target of interest and sparing any neighboring tissues as much as possible. For s.c. tumors, this generally consisted of 2 opposed 1 cm beams targeting the tumor and tangential to the skin surface. For oral treatments, this generally consisted of 2 opposed lateral 1 cm beams targeting the mouth and avoiding the brain. Prior to treatment, each treatment plan was constructed and validated using radiochromic film measurement in a tissue-like phantom (42).

### m^6^A ELISA.

Total RNA was extracted from cells using RNeasy Plus Mini Kit (74134, Qiagen). RNA concentration was measured by NanoDrop 2000 (Thermo Fisher Scientific). A total of 200 ng RNA was used for this experiment. Global m^6^A was measured with m^6^A RNA Methylation Quantification Kit (P-9005-96, EpiGentek) according to manufacturer instruction.

### Statistics.

For comparisons of 2 groups, a 2-tailed unpaired Student’s *t* test was performed. Statistical analyses were performed using GraphPad Prism 10.0 and SAS v9.4. Generalized linear models with fixed effects for study day, treatment group, and group-by-day interaction and random effects for mouse were used to test for differences between groups over time. Tests were 2 sided, and *P* < 0.05 was considered statistically significant.

### Study approval.

For experiments in mouse models, all procedures for mice were approved by the IACUC of Stanford University in accordance with the institutional and NIH guidelines.

### Data availability.

The data included in this article, along with supplementary information and supporting data, can be accessed online or requested from the corresponding author. The raw data are available in the Supporting Data Values file.

## Author contributions

EBR conceived and supervised the study. EBR, QTL, EEG, RLF, CK, KMC, JMN, and LJ designed the experiments and interpreted the data. LJ, LP, JW, HC, SM, SS, LG, EEL, RVE, and SAR performed the experiments. LJ and EBR wrote and revised the manuscript. All other authors read the manuscript and provided comments.

## Supplementary Material

Supplemental data

Unedited blot and gel images

Supporting data values

## Version 1 06/09/2025

Electronic publication
