# Supplementary material for: FTO inhibition enhances the therapeutic index of radiation therapy in head and neck cancer
Source: JCI Insight. 2025 Jun 9;10(11):e184968. doi: 10.1172/jci.insight.184968 (PMC12220955; doi:10.1172/jci.insight.184968)
Supplement: Supplemental data [file jciinsight-10-184968-s198.pdf]

Supplemental Materials for

FTO inhibition enhances the therapeutic index of radiation therapy in head and neck cancer.

Lu Ji1, Leighton Pu[1], Jinglong Wang[1], Hongbin Cao[1], Stavros Melemenidis[1], Subarna Sinha[2], Li Guan[1], Eyiwunmi Eghonghon Laseinde[1], Rie von Eyben[1], Sara Richter[1], Jin-Min Nam[3,4], Christina S Kong[5], Kerriann M Casey[6], Edward E Graves[1], Richard L Frock[1], Quynh Thu Le[1], Erinn B Rankin[1,7]

1Department of Radiation Oncology, Stanford University, Stanford, CA, United States
2Department of Computer Science, Stanford University, Stanford, CA, United States
3Division of Systemic Life Science, Graduate School of Biostudies, Kyoto University, Japan
4Global Center for Biomedical Science and Engineering, Hokkaido University, Japan
5Department of Pathology, Stanford University, Stanford, CA, United States
6Department of Comparative Medicine, Stanford University, Stanford, CA, United States
7Department of Obstetrics and Gynecology, Stanford University, Stanford, CA, United States

*Corresponding author: Erinn B. Rankin, PhD
269 Campus Drive
1245A CCSR
Stanford, CA 94305
E-mail: erankin@stanford.edu
Tel: 650-497-8742

Supplementary Table 1. siRNAs used in this paper

siRNA	Company	Identifier	
**ON-TARGETplus non-targeting siRNA#1**	Horizon Discovery	D-001810-01-05	Pooled
**ON-TARGETplus Human FTO siRNA**	Horizon Discovery	L-004159-01-0010	Pooled
**ON-TARGETplus Mouse Fto siRNA**	Horizon Discovery	L-062238-01-0005	Pooled

Supplementary Table 2. Plasmids used in this paper

Plasmid	Company	Identifier/Clone	Sequence
**Dox-inducible non-targeting shScr**	Dharmacon	VSC11653	
**Dox-inducible human shFTO**	Dharmacon	V3SH11252-227508210	
**Human shFTO #3**			CCCATTAGGTGCCCATATTTA
**Human FTO WT**	GeneCopoeia	EX-H1661-Lv201	
**Human FTO mut**	GeneCopoeia	EX-H1661-Lv201	Mutations: H231A and D233A
**shRNA Control plasmid**	Millipore Sigma	SHC002	
**Mouse shFto #1**	Millipore Sigma	TRCN0000183897	CCAGGGAGACTGCTATTTCAT
**Mouse shFto #2**	Millipore Sigma	TRCN0000277193	GTCTCGTTGAAATCCTTTGAT

Supplementary Table 3. Antibodies used in this paper

Antibody	Company	Identifier	Dilution
**FTO (D2V1I) Rabbit mAb**	Cell Signaling Technology	45980S	1:1000 for WB
**RAD51 (Ab-1) Rabbit pAb**	EMD Millipore	PC130-100UL	1:1000 FOR WB; 1:500 for IF
**Phospho-Histone H2A.X (Ser139) Rabbit mAb**	Cell Signaling Technology	9718S	1:500 for IF
**β-Actin (13E5) Rabbit mAb (HRP Conjugate)**	Cell Signaling Technology	#5125	1:5000 for WB

**Goat anti Rabbit IgG (H+L) Secondary Antibody, Alexa Fluor 594**	Invitrogen	A-11012	1:1000 for IF

Supplementary Table 4. Primers and oligos used in this paper

Primer	Forward	Reverse
**β-actin**	CACCATTGGCAATGAGCGGTTC	AGGTCTTTGCGGATGTCCACGT
**FTO**	TCACCAAGGAGACTGCTATTT	TCACCAAGGAGACTGCTATTT
**Oligos**	**Sequence**	
**EGFP-sgRNA**	ctcgtgaccaccctgaccta	
**BFP donor**	cctgaagttcatctgcaccaccggcaagctgcccgtgccctggcccaccctcgtgaccaccctgaGcCacggGgtgcagtgcttcagccgctaccccgaccacatgaagcagcacgacttcttcaagtccgccatgcc	

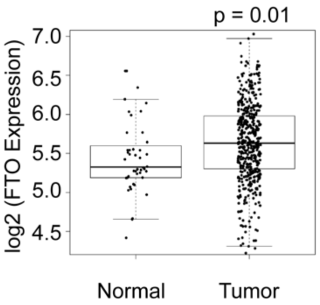

Figure S1. FTO is overexpressed in HNSCC. FTO is overexpressed at the RNA level in HNSCC tumors compared to normal adjacent tissue in TCGA dataset (p=0.01) as determined by the Wilcoxon rank sum test.

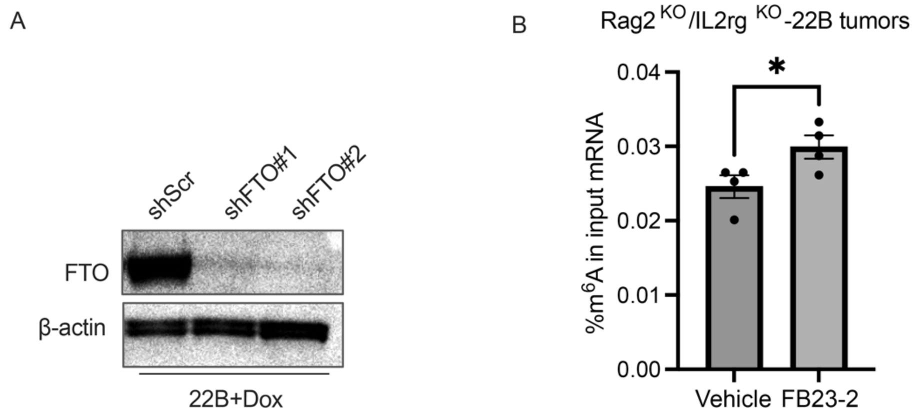

Figure S2. Verification of FTO knockdown in the doxycycline inducible shFTO UM-SCC-22B cell lines and the effect of FB23-2 on global m^{6}A RNA levels in HNSCC tumors. (**A**) Western blot analysis of FTO expression in doxycycline inducible control (shScr) and FTO knockdown (shFTO#1 and shFTO#2) UM-SCC-22B cells 3 days after doxycycline treatment. (**B**) Percentage of total m^{6}A RNA levels are increased in UM-SCC-22B tumors collected from mice treated with FB23-2 (4.6 mg/kg for 5 days) as determined by ELISA (n= 4 per group). $*p < 0.05$ as determined by two-tailed Student's t-test. Data are presented as mean ± SEM.

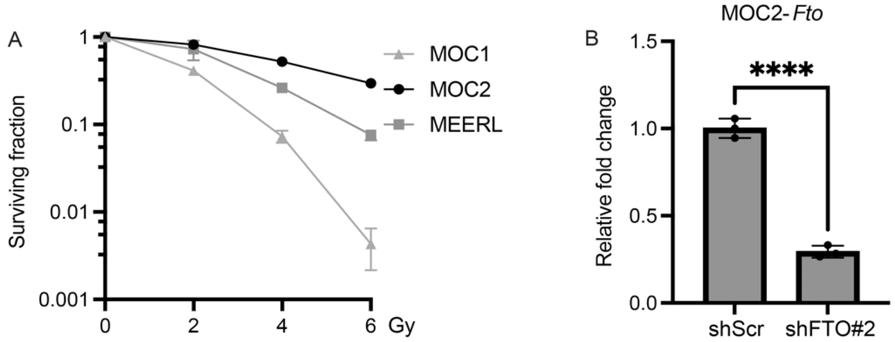

Figure S3. MOC2 is a radiation resistant murine HNSCC cell line. (**A**) MOC2 cells has the highest surviving fraction compared to MOC1 and MEERL cells as determined by clonogenic assay. (**B**) Real time PCR analysis of *Fto* mRNA expression in stable shRNA control (shScr) and FTO knockdown (shFTO) MOC2 cells. In all relevant panels, ****p < 0.0001 as determined by two-tailed Student's t-test. Data are presented as mean ± SEM.

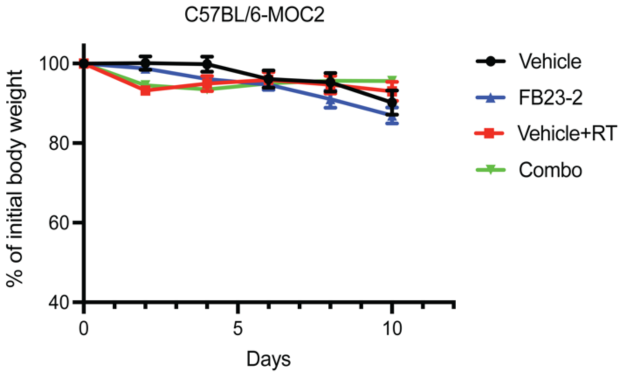

Figure S4. FB23-2 treatment does not reduce body weight in mice bearing MOC2 tumors. Percentage of initial body weight in MOC2 tumor bearing mice in the vehicle (n=13), FB23-2 (4.6 mg/kg daily, n=13), vehicle+ RT (10 Gy RT, n=12), or FB23-2 (4.6 mg/kg and 10 Gy RT, n=12).

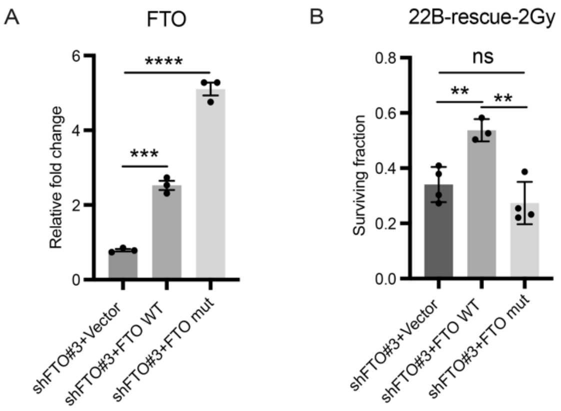

Figure S5. FTO expression enhances the clonogenic growth and survival of irradiated FTO knockdown HNSCC cells. (A) Relative fold change in FTO mRNA expression in shFTO#3 UM-SCC-22B cells with ectopic expression of the control vector (Vector), wild type FTO (FTO WT) or a FTO demethylase mutant (FTO mut). **(B)** Ectopic expression of wild type FTO (FTO WT) promotes clonogenic growth and survival of irradiated (2 Gy) shFTO#3 knockdown cells. Surviving fraction of shFTO#3 UM-SCC-22B cells after 2 Gy irradiation. In all relevant panels, $**p < 0.01$; $***p < 0.001$; $****p < 0.0001$ as determined by two-tailed Student's t-test and two-way ANOVA test. Data are presented as mean ± SEM.

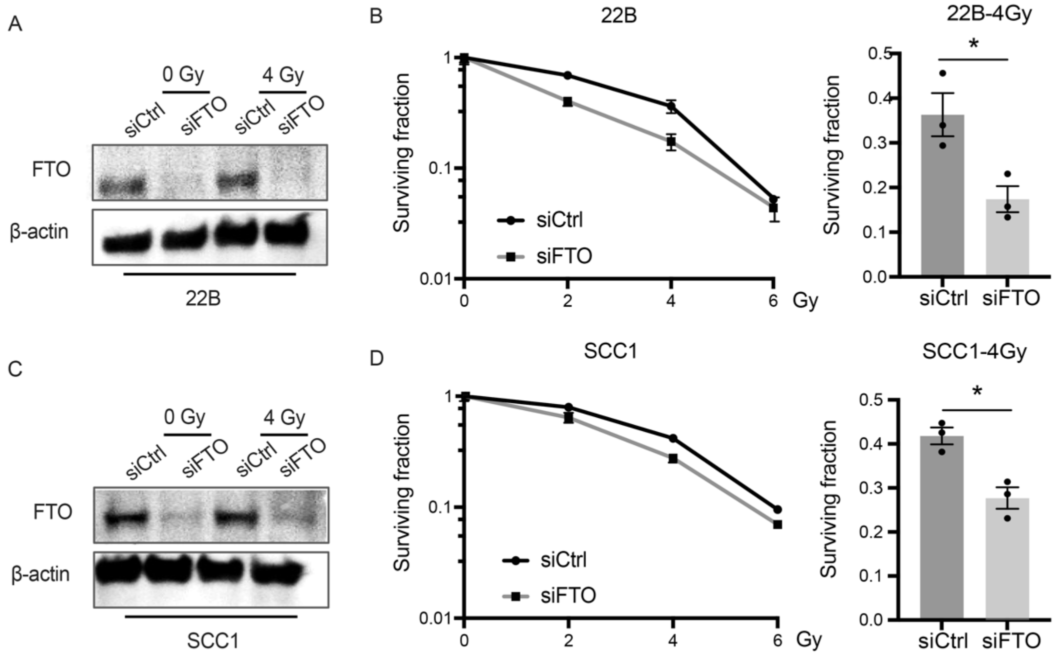

Figure S6. siRNA mediated FTO knockdown in human HNSCC cells enhances radiosensitivity. (**A** and **C**) Western blot analysis of FTO expression in siRNA SMARTpool nontargeting control (siCtrl) and FTO targeting (siFTO) UM-SCC-22B (**A**) and SCC1 (**C**) cells 24 hours after 0 Gy or 4 Gy irradiation. (**B** and **D**) Effect of FTO knockdown on UM-SCC-22B (**B**) and SCC1 (**D**) clonogenic survival and radiation sensitivity (0, 2, 4, and 6 Gy; left panel). The surviving fraction is normalized to its control (0 Gy) and statistically compared to the control (siCtrl) group at 4 Gy (right panel, n=3). Data are presented as mean ± SEM; $*p < 0.05$ as determined by two-tailed Student's t-test (**B** and **D**).

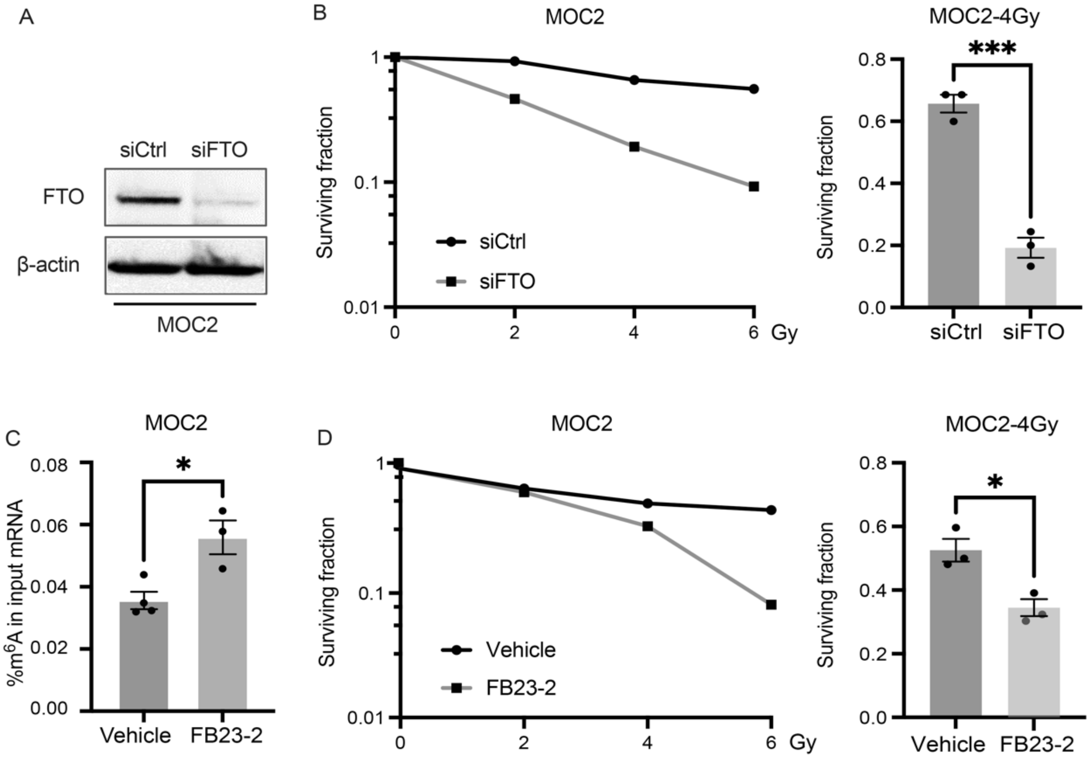

Figure S7. FTO inhibition promotes radiosensitivity in murine HPV negative HNSCC cells. (**A**) Western blot analysis of FTO expression in siRNA SMARTpool nontargeting control (siCtrl) and murine FTO targeting (siFTO) MOC2 cells 2 days after siRNA transfection. (**B**) Effect of FTO knockdown on MOC2 clonogenic survival and radiation sensitivity (0, 2, 4, and 6 Gy; left panel). The surviving fraction is normalized to its control (0 Gy) and statistically compared to the control (siCtrl) group at 4 Gy (right panel, n=3). (**C** and **D**) Pharmacologic inhibition of FTO enhances the radiation response in murine HPV-negative HNSCC cells. (**C**) The percentage of total m^{6}A RNA in FB23-2 treated MOC2 cells 24 hours after FB23-2 treatment (5 μM). (**D**) The effect of FB23-2 treatment on MOC2 clonogenic survival and radiation sensitivity (0, 2, 4, and 6 Gy; left panel). The surviving fraction is normalized to its control (0 Gy) and statistically compared to the control (vehicle) group at 4 Gy (right panel, n=3). Data are presented as mean ± SEM; $*p < 0.05$, $***p < 0.001$ as determined by two-tailed Student's t-test.

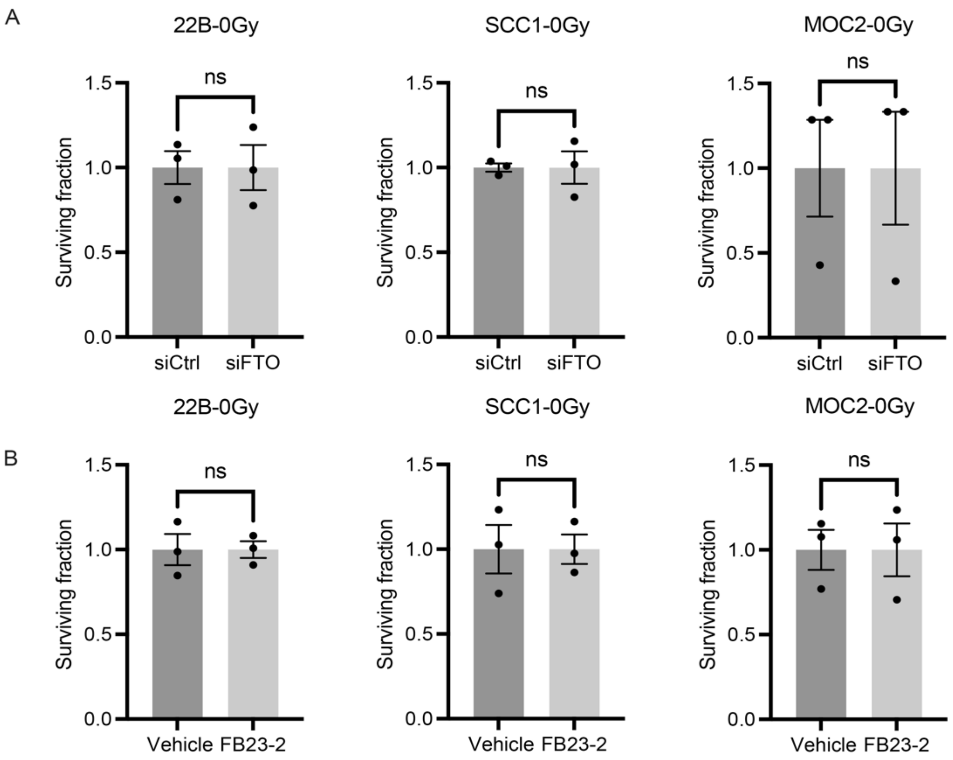

Figure S8. FTO inhibition does not reduce HNSCC clonogenic survival at 0 Gy. (**A**) Genetic inactivation of FTO does not reduce HNSCC clonogenic survival at 0 Gy. Effect of FTO siRNA knockdown on UM-SCC-22B, SCC1 and MOC2 clonogenic survival at 0 Gy. (**B**) Pharmacologic inhibition of FTO does not reduce HNSCC clonogenic survival at 0 Gy. Effect of FB23-2 treatment on UM-SCC-22B, SCC1 and MOC2 clonogenic survival at 0 Gy. The surviving fraction is normalized to its control (0 Gy) and statistically compared to the control (siCtrl or vehicle) group at 0 Gy (n=3). Data are presented as mean ± SEM; ns, not significant as determined by two-tailed Student's t-test.

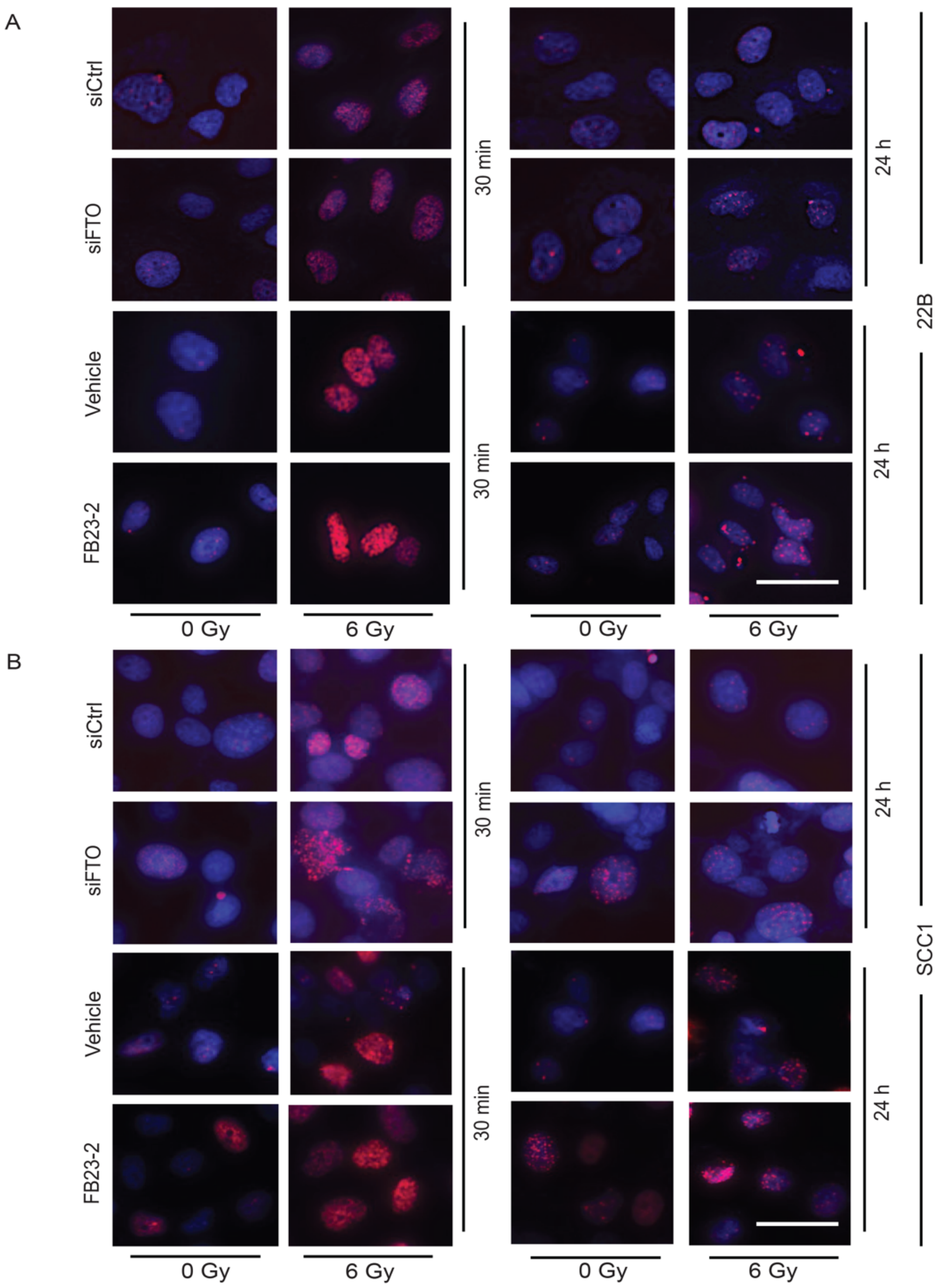

Figure S9. FTO inhibition results in persistent DNA damage post irradiation. (**A** and **B**) Genetic and pharmacologic inhibition of FTO increases γH2AX foci at 24 hours post irradiation in human HPV-negative HNSCC cells. A and B show representative images of γH2AX foci (red) immunofluorescence staining at 30 minutes and 24 hours post irradiation in vehicle, FB23-2, siControl and siFTO UM-SCC-22B (**A**) and SCC1 (**B**) cells. Nuclei were stained with DAPI (blue). Scale bars: 25 μm.

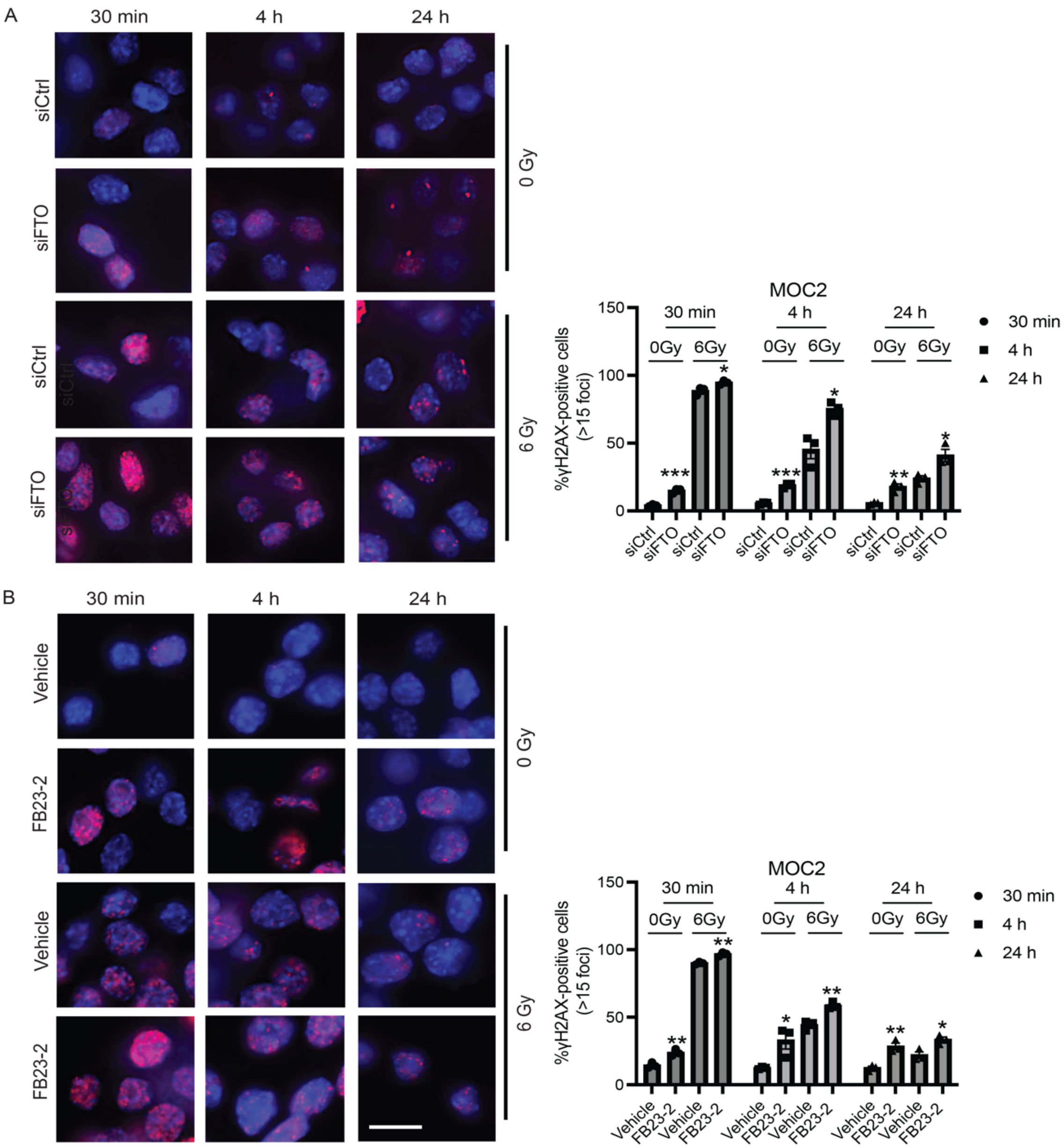

Figure S10. FTO inhibition results in increased DNA damage in irradiated murine HPV negative HNSCC cells. (**A** and **B**) Inhibition of FTO increases γH2AX foci in MOC2 murine HPV-negative HNSCC cells. A and B show representative images of γH2AX foci (red) immunofluorescence at 30 minutes, 4 hours and 24 hours post 0 Gy or 6 Gy irradiation; and quantification of γH2AX foci (red) immunofluorescence staining 30 minutes, 4 hours and 24 hours post irradiation in siRNA (SMARTpool) control and FTO knockdown MOC2 (**A**) and vehicle and FB23-2 (5 μM) treated MOC2 (**B**) cells. Quantification of the percentage of cells with > 15 γH2AX foci per cell is based on 3 random fields and 30 cells from each biologic replicate (n=3). Each dot represents the average for an individual biologic replicate in each group. Nuclei were stained with DAPI (blue). Scale bars: 10 μm. In all relevant panels, $*p < 0.05$; $**p < 0.01$; $***p < 0.001$ as determined by two-tailed Student's t-test and two-way ANOVA test. Data are presented as mean ± SEM.

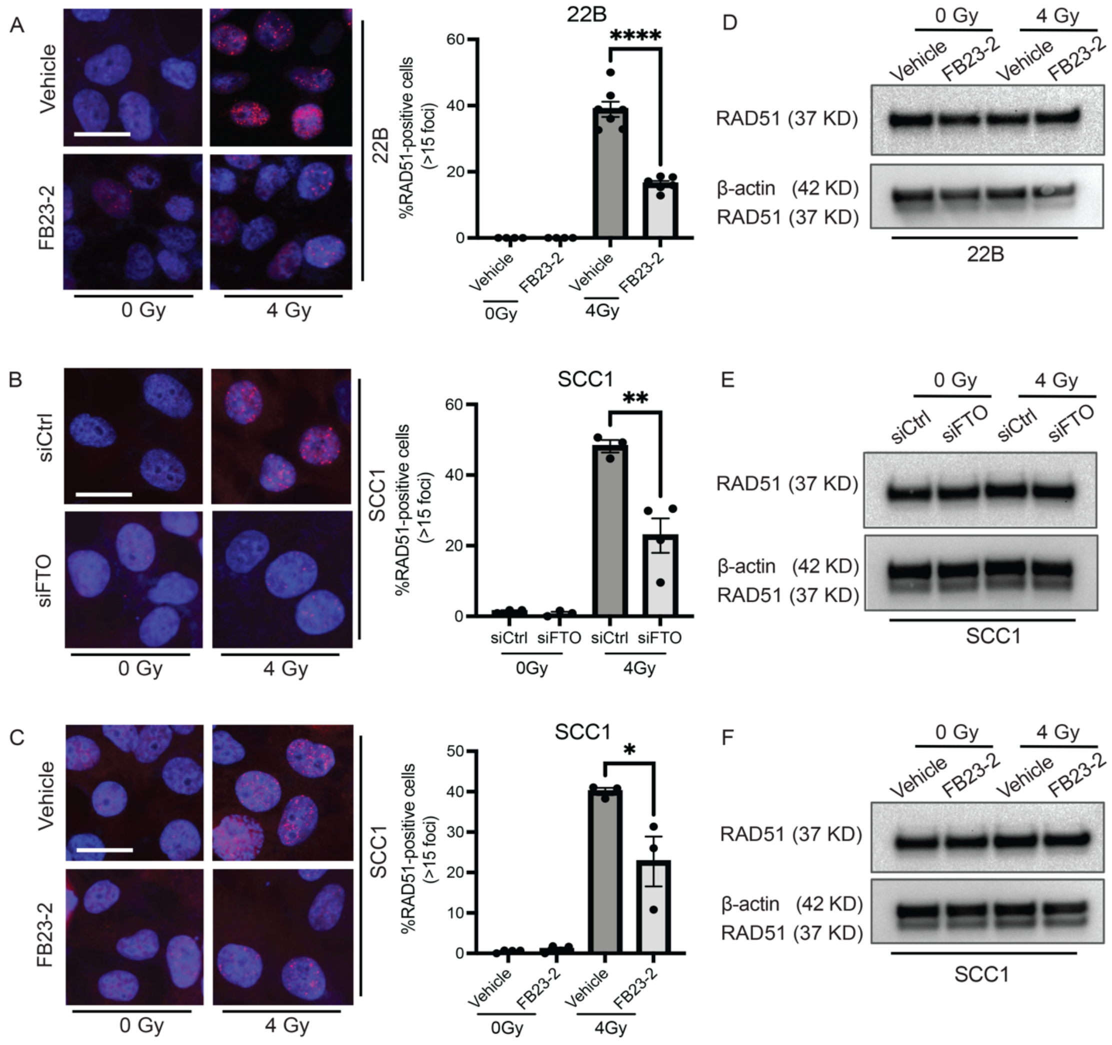

Figure S11. FTO inhibition reduces RAD51 foci formation in irradiated human HNSCC cells. (**A-C**) Representative images (left) and quantification of the percentage of cells with >15 RAD51 foci per cell based on 3 random fields and 30 cells from each biologic replicate (n=3-5, right) of RAD51 foci (red) formation in vehicle and FB23-2 (5 uM) treated UM-SCC-22B cells (**A**), siRNA control (siCtrl) and FTO knockdown (siFTO) SCC1 (**B**) and vehicle and FB23-2 (5 uM) treated SCC1 cells (**C**) at 4 hours post 0 Gy or 4 Gy irradiation. Scale bar = 25 μm. Nuclei were stained with DAPI (blue). (**D-F**) Western blot analysis of RAD51 protein levels in all cell lines described above. In all relevant panels, data are presented as mean ± SEM; $*p < 0.05$, $**p < 0.01$, $****p < 0.0001$ as determined by two-tailed Student's t-test and two-way ANOVA.
