# Supplementary material for: FTO inhibition enhances the therapeutic index of radiation therapy in head and neck cancer
Source: JCI Insight. 2025 Jun 9;10(11):e184968. doi: 10.1172/jci.insight.184968 (PMC12220955; doi:10.1172/jci.insight.184968)

Full unedited gel for Figure 3A

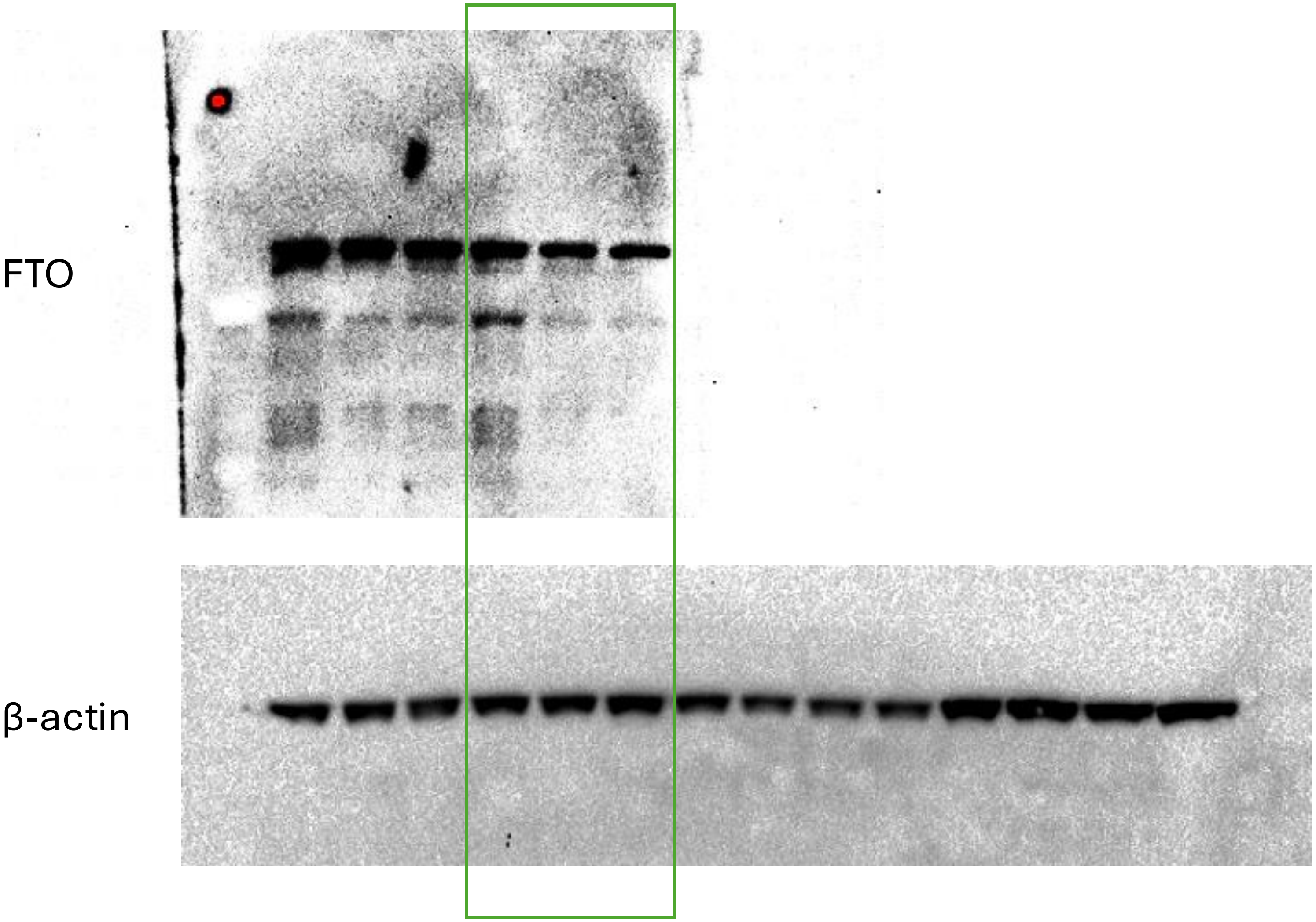

Full unedited gel for Figure 3C

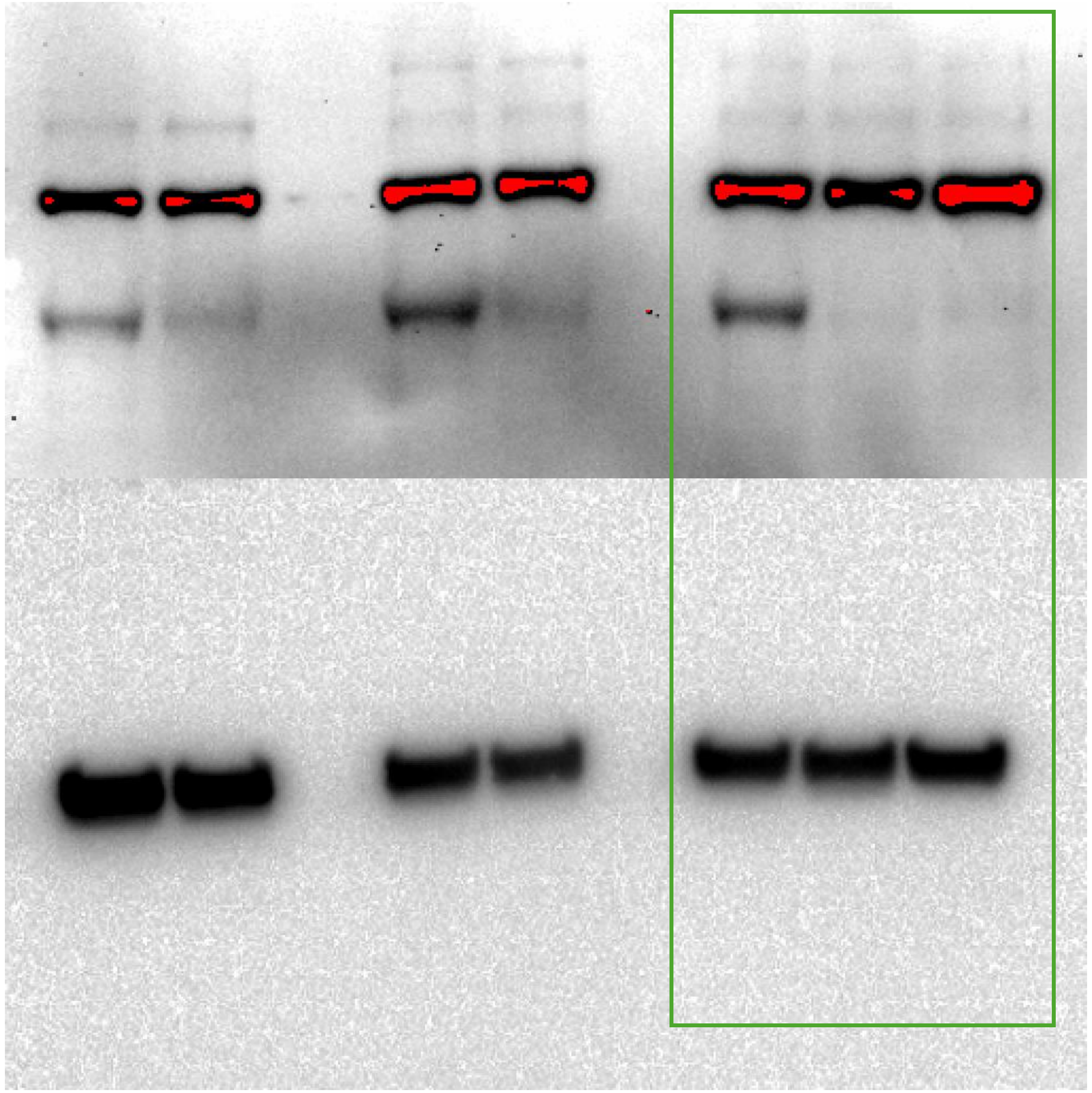

Full unedited gel for Figure 5B

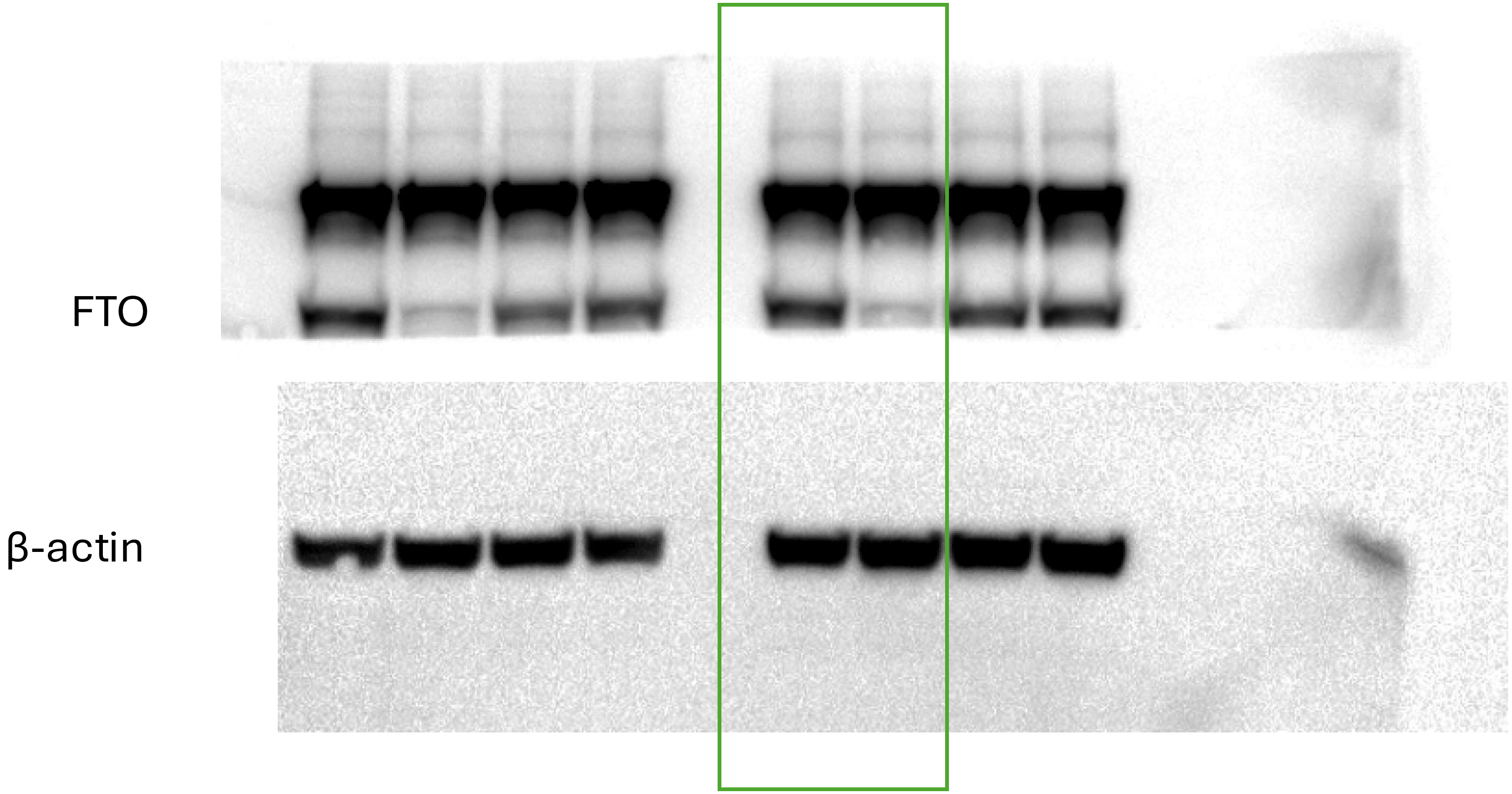

Full unedited gel for Figure 5F

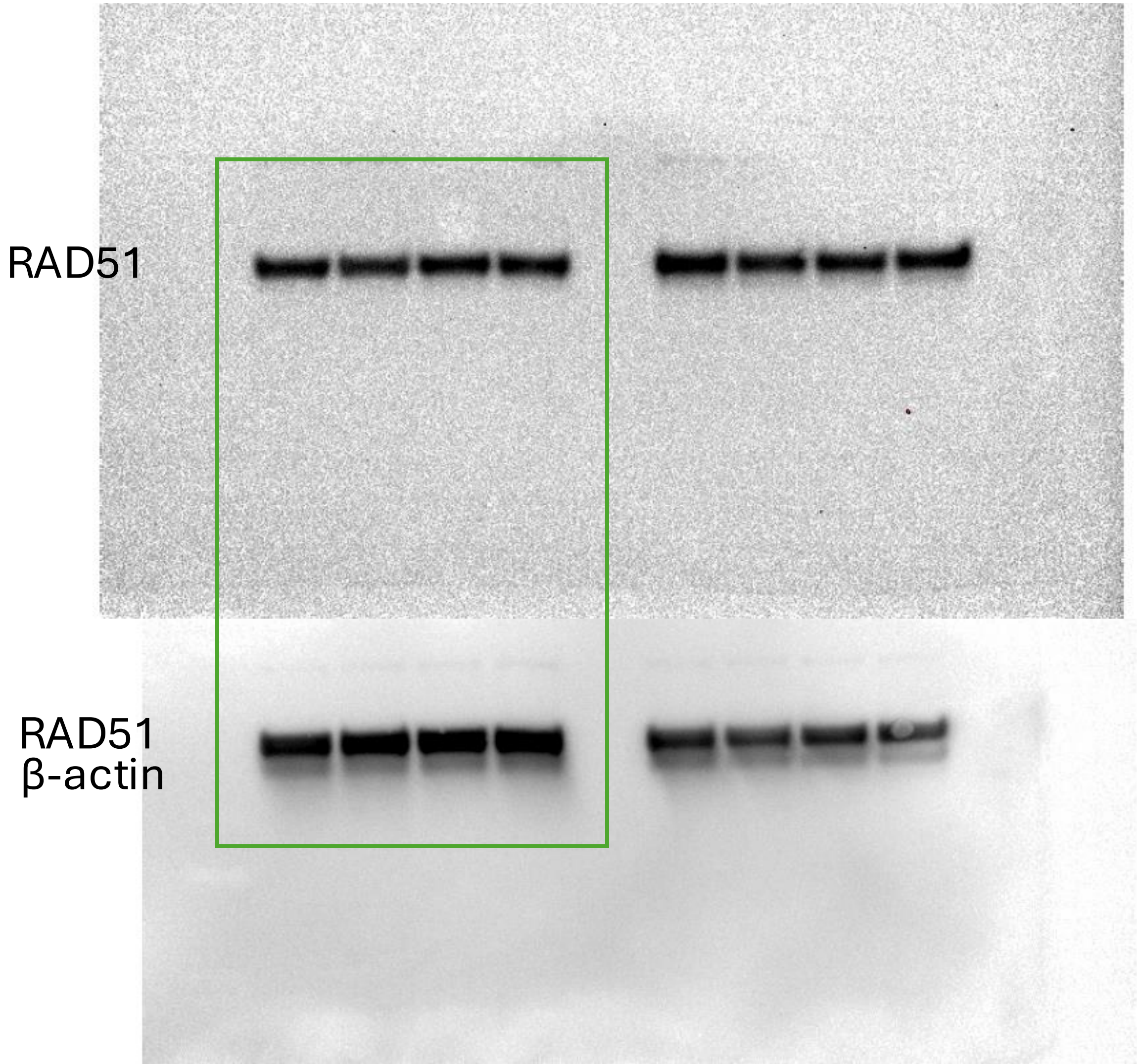

Full unedited gel for Figure S2A

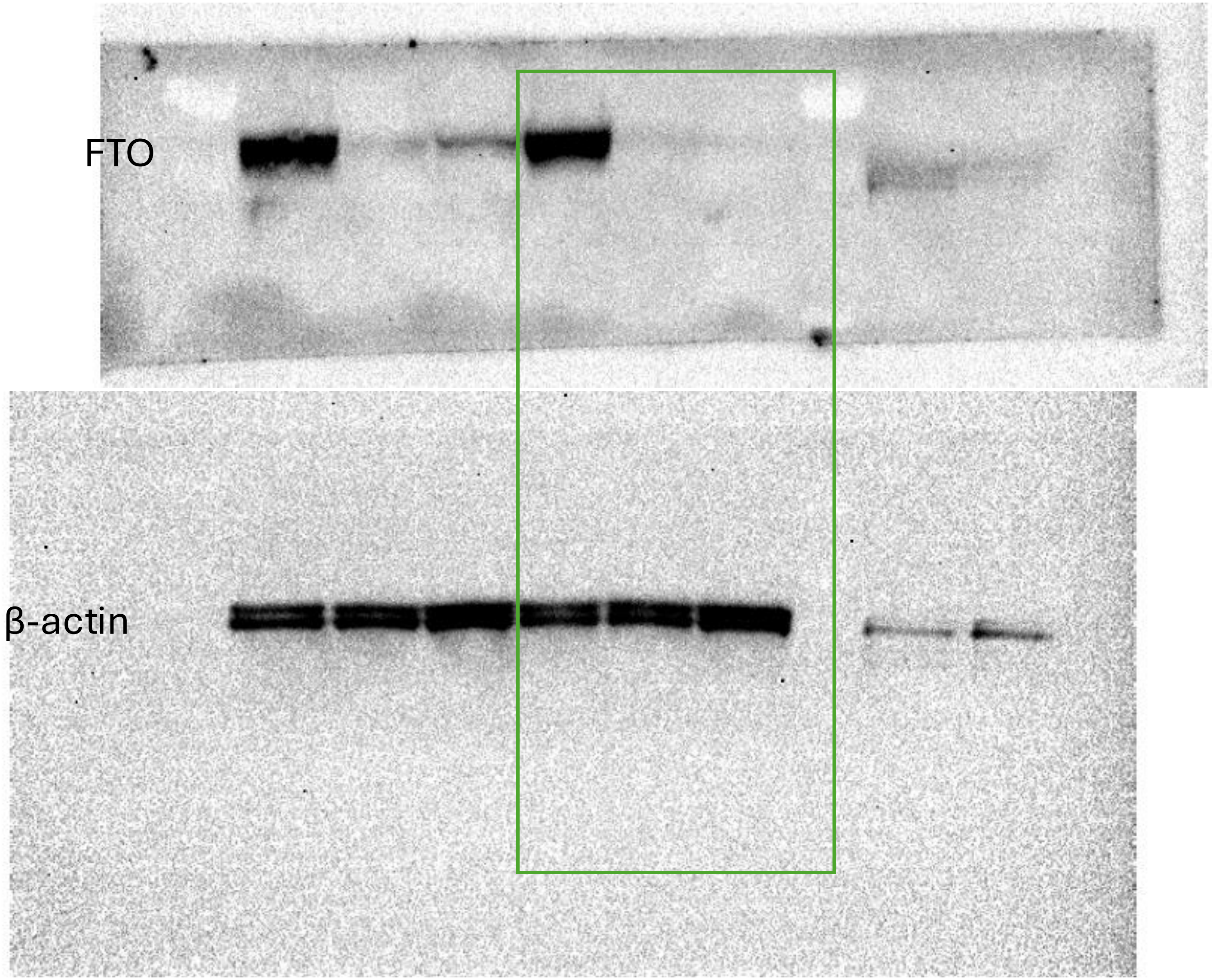

Full unedited gel for Figure S6A

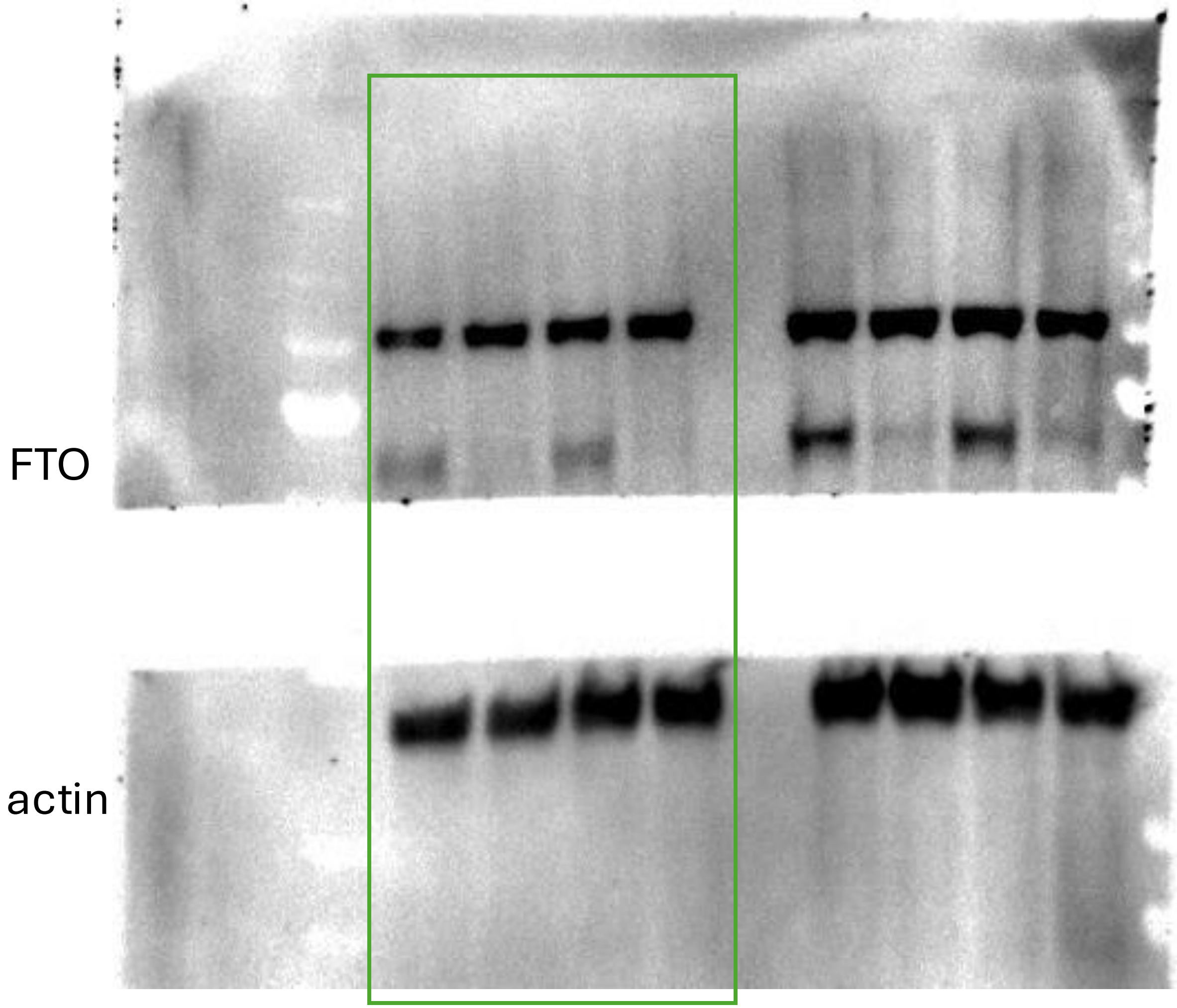

Full unedited gel for Figure S6C

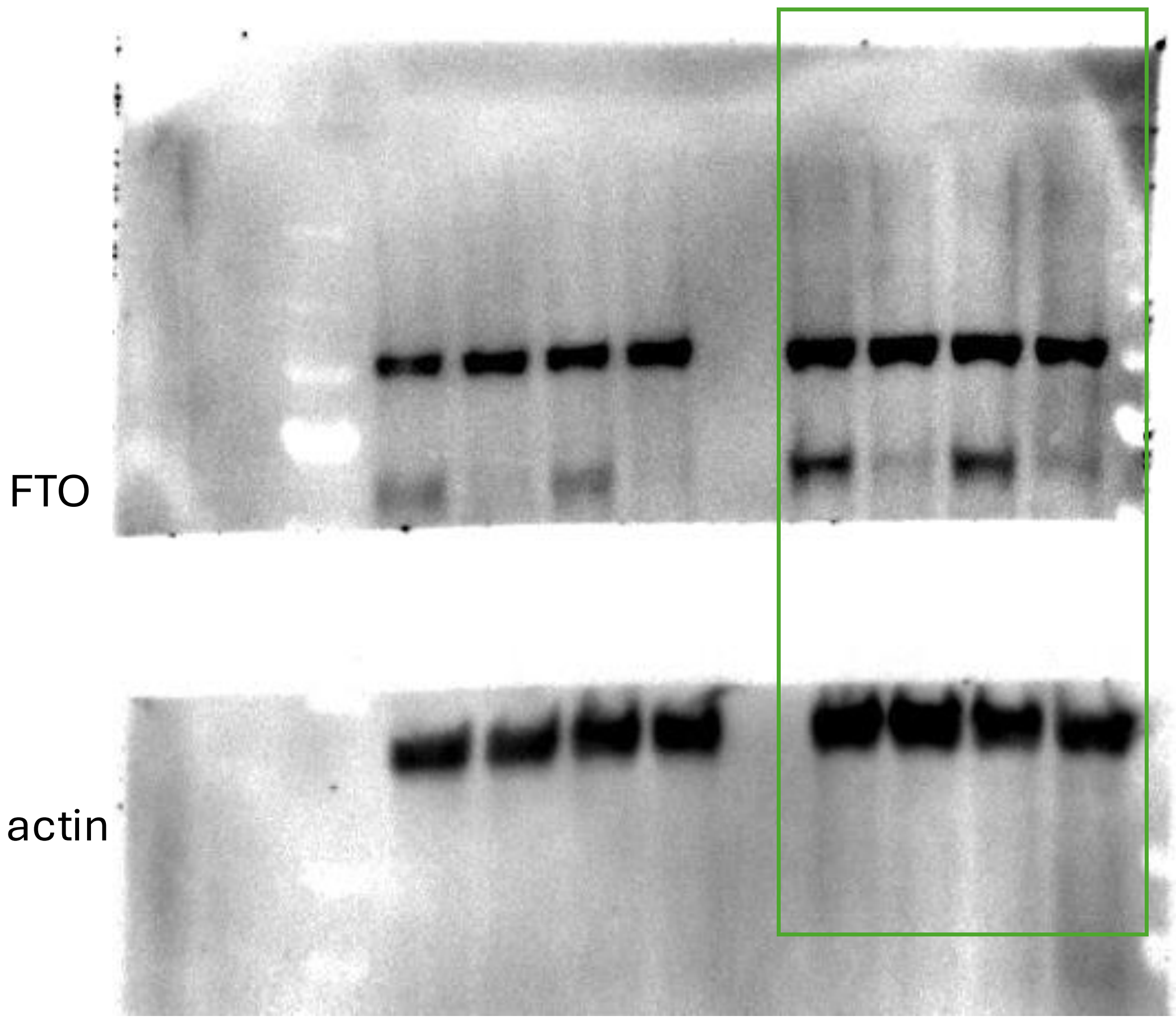

Full unedited gel for Figure S7A

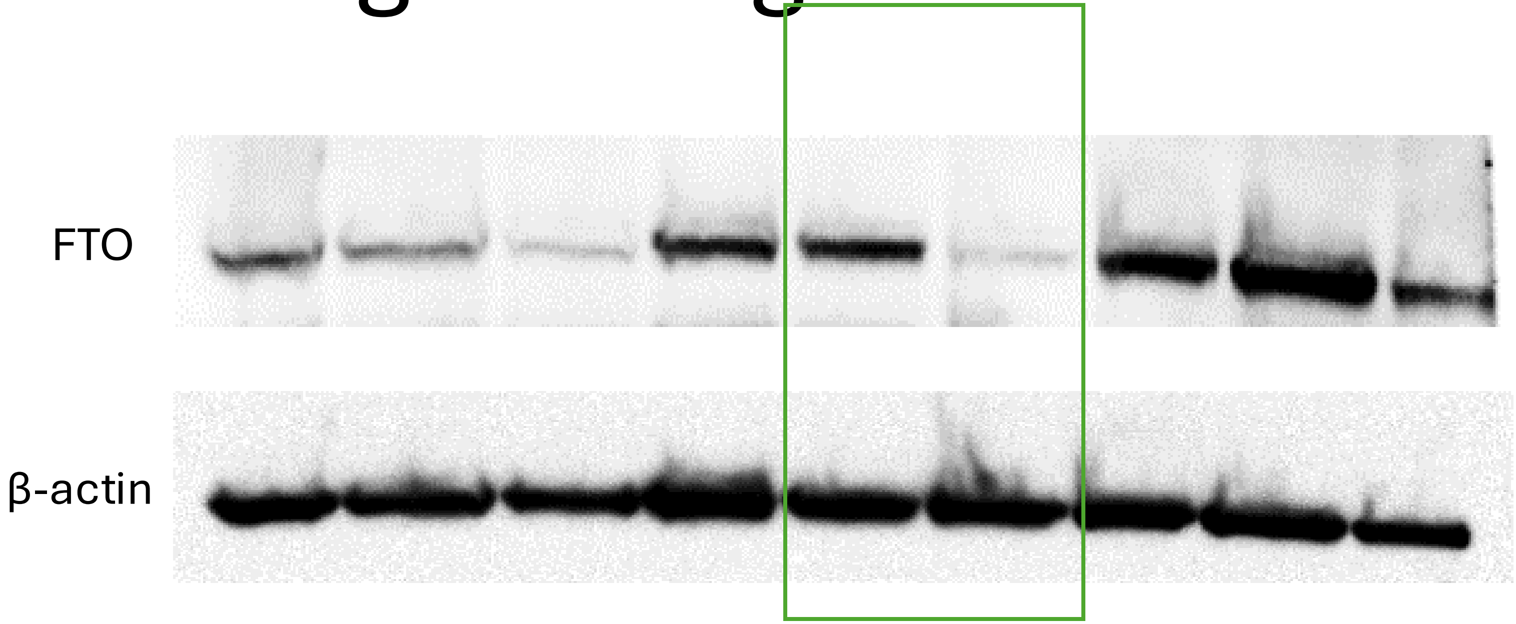

Full unedited gel for Figure S11D

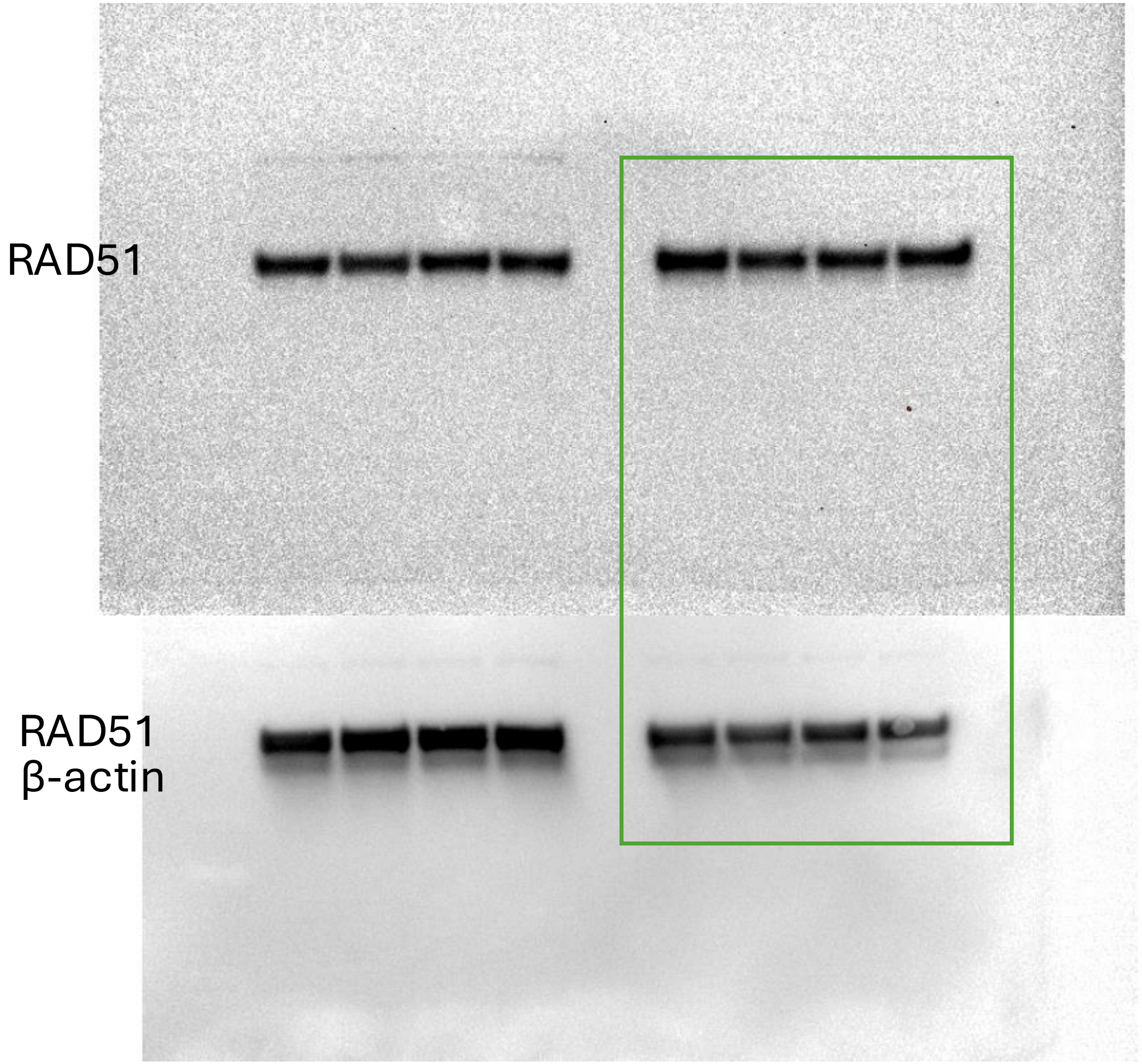

Full unedited gel for Figure S11E

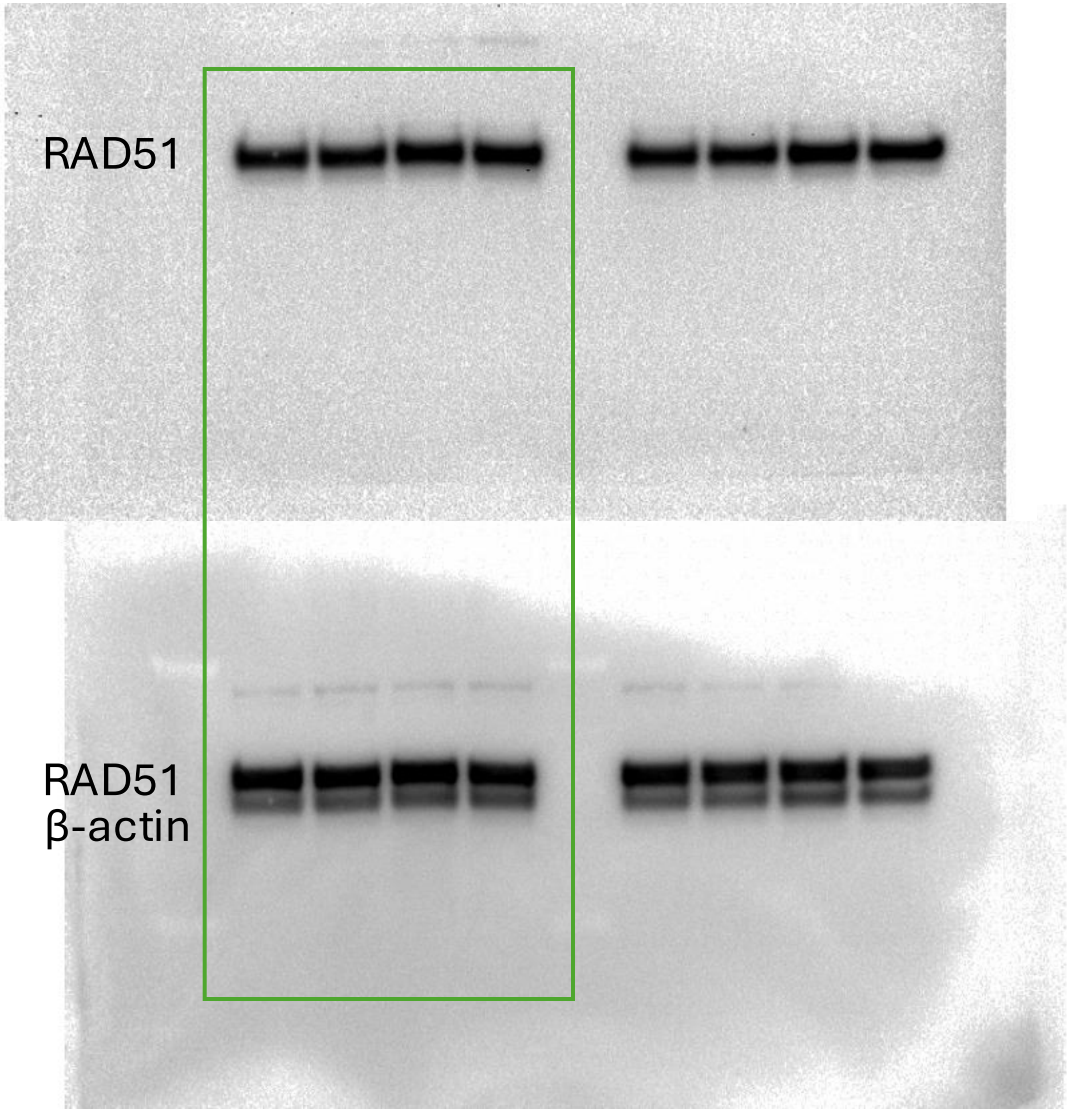

Full unedited gel for Figure S11F

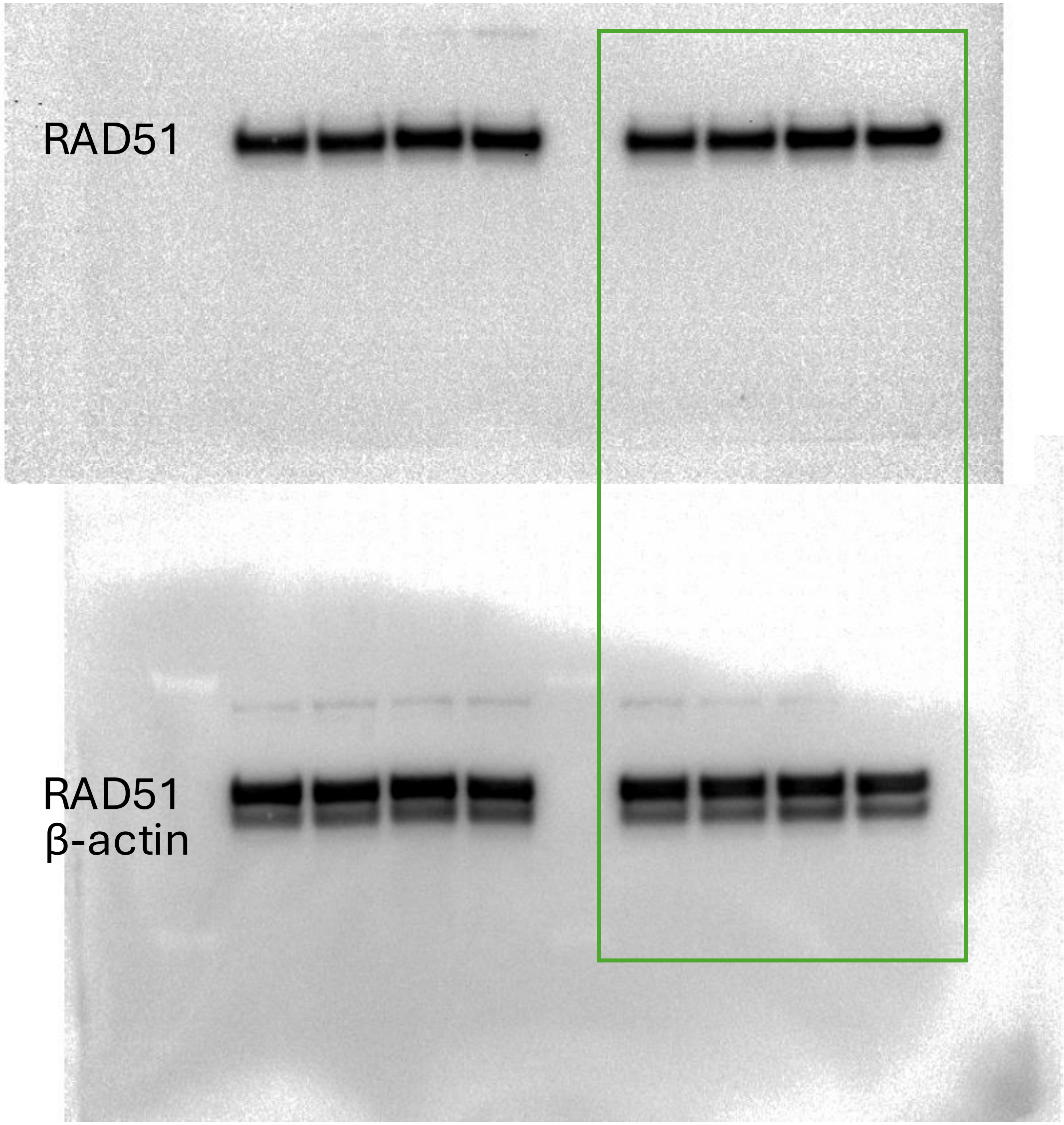

Supplement: Unedited blot and gel images [file jciinsight-10-184968-s199.pdf]
